# Statins Enhance the Molecular Response in Chronic Myeloid Leukemia when Combined with Tyrosine Kinase Inhibitors

**DOI:** 10.3390/cancers13215543

**Published:** 2021-11-04

**Authors:** Hyeok-Jae Jang, Young-Min Woo, Kazuhito Naka, Jong-Ho Park, Ho-Jae Han, Hee-Jin Kim, Sun-Hee Kim, Jae-Sook Ahn, Taehyung Kim, Shinya Kimura, Sarah Zarabi, Jeffrey H. Lipton, Mark D. Minden, Chul-Won Jung, Hyeoung-Joon Kim, Jong-Won Kim, Dennis Dong Hwan Kim

**Affiliations:** 1 Department of Health Sciences and Technology, Samsung Advanced Institute for Health Sciences and Technology, Sungkyunkwan University, Seoul 06351, Korea; nasty27@skku.edu (H.-J.J.); wymirg@skku.edu (Y.-M.W.); hojae.han@sbri.co.kr (H.-J.H.); 2 Department of Stem Cell Biology, Research Institute for Radiation Biology and Medicine, Hiroshima University, Hiroshima 734-8553, Japan; kanaka55@hiroshima-u.ac.jp; 3 Clinical Genomics Center, Samsung Medical Center, Seoul 06351, Korea; jongho11.park@samsung.com; 4 Department of Laboratory Medicine and Genetics, Samsung Medical Center, Sungkyunkwan University School of Medicine, Seoul 06351, Korea; hee_jin.kim@samsung.com (H.-J.K.); sunnyhk.kim@samsung.com (S.-H.K.); 5 Department of Hematology/Oncology, Chonnam National University School of Medicine, Hwasun 58128, Korea; ahnjaesook@hanmail.net (J.-S.A.); hjoonk@chonnam.ac.kr (H.-J.K.); 6 Department of Computer Science, University of Toronto, Toronto, ON M5S 2E4, Canada; taehyung.kim@mail.utoronto.ca; 7 The Donnelly Centre for Cellular and Biomolecular Research, University of Toronto, Toronto, ON M5S 3E1, Canada; 8 Department of Medical Oncology & Hematology, Saga University School of Medicine, Saga 840-8502, Japan; shkimu@cc.saga-u.ac.jp; 9 Department of Medical Oncology & Hematology, Princess Margaret Cancer Centre, University Health Network, University of Toronto, Toronto, ON M5G 2M9, Canada; sarah.zarabi@mail.utoronto.ca (S.Z.); jeff.lipton@uhn.ca (J.H.L.); minden@uhnres.utoronto.ca (M.D.M.); 10 Ontario Cancer Institute, University of Toronto, Toronto, ON M5G 2M9, Canada; 11 Department of Hematology/Oncology, Samsung Medical Center, Sungkyunkwan University School of Medicine, Seoul 06351, Korea; chulwon1.jung@samsung.com

**Keywords:** CML, drug resistance, statin, tyrosine kinase inhibitor, combination therapy

## Abstract

**Simple Summary:**

Approximately 50–60% of patients with chronic myeloid leukemia (CML) achieve a stable deep molecular response (DMR) after tyrosine kinase inhibitor (TKI) therapy. The achievement of DMR is a prerequisite for treatment-free remission. Repurposing statins is a straightforward strategy for enhancing molecular response in CML treatment. Second-generation TKIs have been reported to exhibit cardiovascular toxicity. Thus, statins have been widely prescribed for patients with CML undergoing second-generation TKI therapy for modifying cardiovascular risk factors, such as hyperlipidemia. Furthermore, the results of this study support the therapeutic benefit of the concomitant use of statins in TKI therapy for patients with CML. Additionally, the potential additive effects of statins and TKIs enhance the DMR rate in patients with CML, rendering these effects clinically relevant in these patients. In particular, this combination is a strong candidate for the achievement of DMR in patients with CML who have not achieved DMR with TKI therapy alone.

**Abstract:**

Previous studies have suggested that statins can be repurposed for cancer treatment. However, the therapeutic efficacy of statins in chronic myeloid leukemia (CML) has not yet been demonstrated. In this study, we retrospectively evaluated the outcomes of 408 CML patients who underwent imatinib therapy. The deep molecular response rates in patients treated with the statin/TKI combination were significantly higher than those in patients treated with TKI alone (*p* = 0.0016). The statin/TKI combination exerted potent cytotoxic effects against wild-type and *ABL1* mutant CML, BaF3, and K562/T315I mutant cells. Furthermore, the statin/TKI combination additively inhibited the colony-forming capacity of murine CML-KLS^+^ cells in vitro. In addition, we examined the additive growth-inhibitory effects of the statin/tyrosine kinase inhibitor (TKI) combination against CML patient-derived CD34^+^ cells. The growth-inhibitory effects of the statin/imatinib combination against CD34^+^/CML primary cells were higher than those against CD34^+^/Norm cells (*p* = 0.005), suggesting that the combination of rosuvastatin and imatinib exerted growth-inhibitory effects against CML CD34^+^ cells, but not against normal CD34^+^ cells. Furthermore, results from RNA sequencing of control and statin-treated cells suggested that statins inhibited c-Myc-mediated and hematopoietic cell differentiation pathways. Thus, statins can be potentially repurposed to improve treatment outcomes in CML patients when combined with TKI therapy.

## 1. Introduction

Chronic myeloid leukemia (CML) is characterized by the presence of the Philadelphia chromosome (Ph) that results from *BCR-ABL1* rearrangement. In the last two decades, advances in tyrosine kinase inhibitor (TKI) therapy have revolutionized the management of CML [1,2]. Consequently, the life expectancy of patients with CML has significantly improved and it is approximately 98% of the life expectancy of the general population [3,4,5]. However, TKI therapy is associated with several side effects and high costs. Hence, several clinical trials have examined the effect of TKI discontinuation in patients with prolonged (more than two years) and deep remissions [6,7,8] with a successful discontinuation rate of approximately 50% without losing leukemia control. Currently, a sustained deep molecular response (DMR) over 2 years or longer is a prerequisite for TKI discontinuation for a treatment-free remission (TFR) attempt, which is defined as a ≥4.0 log reduction (MR^4.0^) in the number of cells with *BCR-ABL1* rearrangement when compared with that in the standard baseline.

Statins, which are HMG-CoA reductase (HMGCR) inhibitors, have been used to treat hypercholesterolemia for decades. The mode of action of statins involves lowering cholesterol levels and improving lipid profiles. Statins reduce the risk of cardiovascular events, including coronary artery disease or stroke, and consequently improve life expectancy in the general population [9,10]. Several studies have suggested that statins can prevent carcinogenesis, potentiate the activities of various antineoplastic agents [11,12], and improve the survival rates of patients with cancer [13,14]. The mechanisms underlying the statin-mediated potentiation of chemotherapy efficacy or improved survival in patients with cancer have not been completely elucidated; however, several mechanisms have been proposed. Statins can trigger tumor-specific apoptosis and growth arrest in several subtypes of leukemia [11]. Statins decrease the expression of the c-Myc protein in ovarian and colorectal cancer cell lines [15]. Additionally, statins inhibit cell proliferation, angiogenesis, and metastasis, which leads to a loss of the self-renewal capacity of stem cells [11,16]. Previous studies have suggested that statins can be repurposed for the treatment of various cancers, including multiple myeloma, breast cancer, and colon cancer [12,17,18]. Although MYC deregulation does not directly confer resistance to imatinib, it might contribute to CML progression through the inhibition of differentiation [19]. However, the therapeutic efficacy of statins in CML has not been previously reported. This study investigated the feasibility of repurposing statins for targeting CD34^+^ cells in CML and consequently enhancing the DMR rate in patients with CML undergoing TKI therapy (Figure S1).

This study aimed to investigate the clinical evidence for an enhanced response rate, especially the DMR rate, in patients with CML after treatment with the statin/TKI combination, as well as the in vitro cytotoxic effects of the statin/TKI combination against CML and the underlying molecular mechanisms.

## 2. Materials and Methods

### 2.1. Analysis of DMR Rates in Patients with CML Who Were Treated with IM Alone or in Combination with a Statin

We evaluated the clinical outcomes of 408 patients with chronic-phase CML to validate the clinical efficacy of the statin/IM combination. The statin group was defined as patients undergoing statin therapy for hypercholesterolemia at the time of IM therapy initiation; we continued the therapy for at least 3 years along with IM therapy. The details of clinical management and disease monitoring using *BCR-ABL* quantitative PCR are summarized in the Supplementary Materials. DMR was evaluated using MR^4.5^ as an endpoint, and other response parameters were also evaluated.

For multivariate analysis, the following variables were considered for modeling: Use of a statin, age (continuous variable), sex, Sokal risk group (low/intermediate-risk vs. high-risk), and additional cytogenetic abnormalities in Ph+ clones. Hazard ratios (HRs) and 95% confidence intervals (CIs) were estimated as significant risk factors based on multivariate analysis. Differences were considered significant at *p* < 0.05.

We performed a propensity score matching (PSM) analysis to compare the treatment outcomes between the statin and non-statin groups, excluding any effect from the potential interaction between the use of statins and other confounding clinical factors. Detailed descriptions of the clinical analysis are provided in the Supplementary Materials. All statistical analyses were performed using R (version 3.2.0; R Foundation for Statistical Computing, Austria) and EZR software [20].

### 2.2. Evaluation of In Vitro Cytotoxicity against CML Cell Lines

K562 and BaF3 cells were seeded in triplicate in 96-well plates (3 × 10^3^ cells/well). Cell viability was examined using the WST-8 assay kit (Dojindo, Japan) at 0, 24, 48, and 72 h post-treatment with statins and/or TKIs. The treated cells were incubated with the WST-8 reagent at 37 °C for 4 h. The absorbance of the mixture at 450 nm (OD_450_) was measured using an xMark microplate absorbance spectrophotometer (Bio-Rad Laboratories, Hercules, CA, USA). The isolated CD34^+^ cells were seeded into individual wells of a 96-well plate (500 cells/well) containing 100 µL of serum-free expansion medium (SFEM) II (Stemcell Technologies, Canada) with the RealTime-Glo MT cell viability assay reagent (Promega, Madison, WI, USA). Cell viability was assessed every 24 h for 8 days using the GloMax-Multi Detection System (Luminometer; Promega). The percentage of viable cells in the treatment group relative to that in the untreated group was determined. To ensure accuracy, more than three independent experiments were performed in triplicate. The phospho-CrkL/CrkL assays were performed as presented in Supplementary Materials.

The following cell lines were used in this study: K562 (obtained from the American Type Culture Collection (ATCC, Manassas, VA, USA) and wild-type or ABL1-mutant BaF3 (wild-type [BaF3/WT] and BaF3/G250E*^mut^*, BaF3/T315I*^mut^*, and BaF3/F317L*^mut^* mutants; provided by Dr. Shinya Kimura of Saga University School of Medicine, Japan). The cells were cultured in the Roswell Park Memorial Institute-1640 medium (Gibco, Carlsbad, CA, USA) or Dulbecco’s Modified Eagle Medium (Gibco) supplemented with 10% fetal bovine serum (Gibco), 100 U/mL penicillin, and 100 µg/mL streptomycin (Gibco).

An isogenic K562 cell line harboring the T315I mutation was generated using the clustered regularly interspaced short repeat (CRISPR)/caspase 9 (Cas9) system with the Cas9 RNA-guided DNA endonuclease. To target the ABL kinase domain within the *BCR-ABL1* fusion gene, a single guide RNA (sgRNA) and two complementary oligo primers were designed. sgRNA was cloned using the Guide-it CRISPR/Cas9 System (Clontech, Palo Alto, CA, USA), according to the manufacturer’s instructions. The clone containing the T315I mutation (c.944C > T; K562/T315I*^mut^*) was selected and validated using capillary sequencing.

Stock solutions of rosuvastatin (hydrophilic properties; Selleckchem, Houston, TX, USA), atorvastatin (lipophilic properties; Selleckchem), imatinib (IM; Gleevec; Novartis, Switzerland), nilotinib (NI; Tasigna; Selleckchem), and dasatinib (DA; Sprycel; Selleckchem) were prepared in dimethyl sulfoxide (DMSO; Sigma-Aldrich, St. Louis, MO, USA) and stored at −20 °C. The drug concentrations were selected on the basis of human pharmacokinetic parameters and preliminary evidence from cell line experiments (Table S1).

### 2.3. Synergy Calculations

We calculated the expected drug combination responses based on the highest single agent (HSA) model and synergy scoring using SynergyFinder 2.0 [21]. Dose–response curves were fitted with a 4-parameter logistic regression (LL4), and readout viability baseline correction was applied.

### 2.4. Colony-Formation Assay

The protocols for the generation of double transgenic mice and the induction of BCR-ABL1 gene expression are described in the Supplemental Materials. All animal care and experimental procedures were performed according to the guidelines for animal and recombinant DNA experiments at Hiroshima University (2019-329 and A20-5). CML *cKit^+^Lineage^−^Sca1^+^* (KLS) cells were isolated from CML mice as described previously [22]. Thereafter, the effect of statins on the colony-forming capacity of CML-KLS cells was determined. Freshly isolated CML-KLS cells were co-cultured with OP-9 stromal cells in the presence of IM (1 µM), DA (0.5 µM), and rosuvastatin (2 µM) or atorvastatin (2 µM) for 72 h. The colonies were counted after 7 days of incubation as previously described [22].

### 2.5. Isolation of Hematopoietic Progenitor Cells from Patients with CML/Healthy Individual

Bone marrow (BM) samples collected from patients with CML (CD34^+^/CML) at the time of initial CML diagnosis were processed. Primary BM CD34^+^ cells (CD34^+^/Norm; PCS-800-012) were obtained from the ATCC. The cells were washed and resuspended in SFEM II at a density of 1 × 10^6^ cells/mL and stained with 5 μg/mL Hoechst 33,342 (Sigma-Aldrich) for 90 min at 37 °C. Next, the cells were incubated with fluorescein isothiocyanate (FITC)-labeled anti-CD34 antibodies (BD Biosciences Pharmingen, San Diego, CA, USA) for 30 min at 4 °C and subjected to flow cytometric analysis. The CD34^+^ cells were isolated as per previously described methods [23]. The purity of CD34^+^ cells, consistently more than 98%, was determined using flow cytometric analysis with a FACSAria III Cell-Sorting System (BD Biosciences, San Jose, CA, USA).

### 2.6. Gene Expression Analysis Using Whole Transcriptome and Targeted RNA Sequencing (RNA-seq)

For gene expression and pathway enrichment analyses, K562 cells were treated with rosuvastatin (1.5 µM) in the presence or absence of IM (0.6 µM) or DMSO (negative control). Whole transcriptome, pathway enrichment, and targeted RNA-seq analyses were performed as described in the Supplementary Materials.

The calculated expression data of 57,773 transcribed genes in the K562 cells belonging to the control, IM single treatment (0.6 µM), rosuvastatin single treatment (1.5 µM), and IM/rosuvastatin combination treatment groups were examined. Differentially expressed gene (DEG) analysis was performed using 33,243 genes that had non-zero raw read counts. The expression data with low read counts were excluded, and the average counts from triplicates of the control and rosuvastatin groups were subjected to upper quantile normalization (<100). Data from 12,061 genes were used for the final analysis.

### 2.7. Targeted RNA-Seq Assay

For further verification, a targeted RNA-seq analysis was performed using a customized assay, which utilizes a molecule-specific barcode—Molecular Indexing—designed to simultaneously analyze 200 genes (BD Biosciences). The K562 cell line (rosuvastatin with or without IM or DMSO) was processed for targeted RNA-seq. Sequencing data deconvolution was performed with an automated algorithm using a Seven Bridges Genomics pipeline (tailor-made for BD Precise generated datasets).

### 2.8. Pathway Enrichment Analysis

Pathway enrichment analysis was performed using both gene ontology enrichment in ConsensusPathDB [24] and DAVID [25] to analyze the molecular function and/or biological processes of the gene classes.

## 3. Results

### 3.1. Clinical Benefits from the Use of Statins in CML Treatment with IM Therapy

In order to evaluate our hypothesis that the use of statin added to TKI therapy in CML treatment could increase the molecular response rate, we performed a retrospective study in 408 CML patients treated with IM therapy at the dose of 400 mg once daily. The study was performed upon the institutional research ethics board’s approval. The responses to IM mesylate therapy were compared according to the concomitant use of statins. The clinical characteristics of the patients are summarized in Table 1, and the treatment outcomes are summarized in the Supplementary Materials. The median follow-up duration was 77 months (range, 6–139 months). The rates of major molecular response (MMR) at 3 years and DMR (defined as MR^4.5^) at 5 years were 65.7 ± 2.5% and 44.2 ± 2.7%, respectively. The MMR and DMR rates did not markedly differ according to other clinical factors.

**Figure 1:**
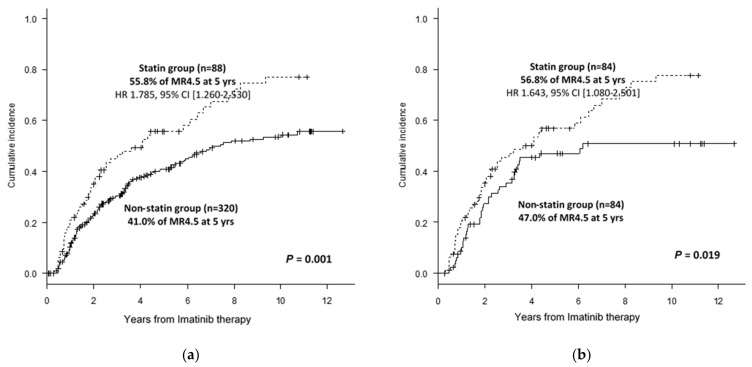
Statins increase the deep molecular response rate in patients with chronic myeloid leukemia (CML) undergoing imatinib therapy. (**a**) Comparison of cumulative incidence of molecular response between the statin group and the non-statin group. (**b**) Comparison of cumulative incidence of molecular response between the statin group and the non-statin group after propensity score matching.

According to the criteria defined for the statin group, 88 patients (21.3%) were categorized in the “statin” group, and 320 patients in the “non-statin” group. The statins administered were atorvastatin (*n* = 44, 50%), rosuvastatin (*n* = 26, 30%), simvastatin (*n* = 10, 11%), pravastatin (*n* = 6, 7%), and fluvastatin (*n* = 2, 2%). The DMR (*p* = 0.0016) and MMR (*p* = 0.0048) rates in the statin group were higher than that in the non-statin group (DMR rates at 5 years, at 55.8% [43.4–66.5%] vs. 41.0% [35.0–47.0%] (Figure 1a); MMR rates at 3 years were 77.3% [65.9–85.3%] vs. 62.5% [56.7–67.9%]).

Multivariate analyses revealed that statin use was an independent clinical factor for DMR and MMR. The concomitant use of statins independently improved the DMR rate by 78.5% (HR 1.785, 95% CI [1.260–2.530], *p* = 0.001). However, other factors, such as Sokal risk, age, sex, or ACAs at presentation, were not identified as independent prognostic factors. Statin use (HR = 1.541; 95% CI = 1.015–2.341; *p* = 0.043), ACAs (HR = 0.381; *p* = 0.0038), and high Sokal risk (HR = 0.687; *p* = 0.042) were independent factors for MMR.

To control for a potential interaction between the use of statin and other clinical factors that may potentially affect the response rate to IM therapy, we applied PSM, and selected 84 case–control pairs (*n* = 168) for further analysis. All pre-treatment variables were well balanced after PSM. Age (*p* = 0.769), Sokal risk group (*p* = 0.486), ACAs (*p* = 0.406), and sex (*p* = 0.440) were not significantly different after PSM. In the propensity score-matched group, the DMR rates in the statin group were higher than that in the non-statin group (*p* = 0.019) (56.8% vs. 47.0% for DMR at 5 years) (Figure 1b). In addition, the statin group showed a trend of higher MMR rates compared to the non-statin group (*p* = 0.073) (78.8% vs. 62.6% for MMR at 3 years). PSM analysis indicated that the concomitant use of statins increased DMR rates even after adjustment for other confounding factors.

### 3.2. Statins Synergistically Potentiate the Cytotoxic Activity of TKIs Against the BCR-ABL1+ Cell Lines

**Figure 2:**
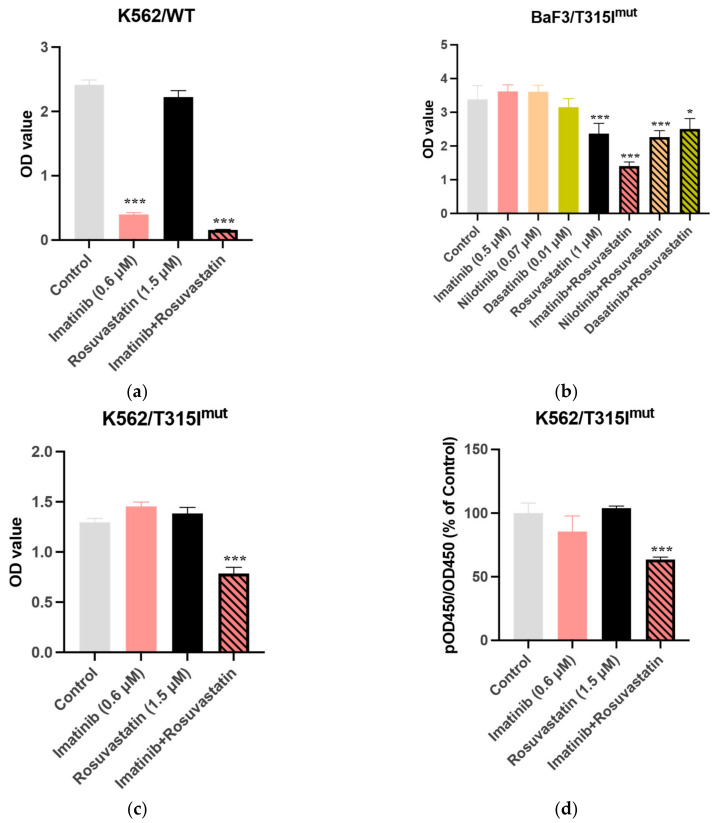

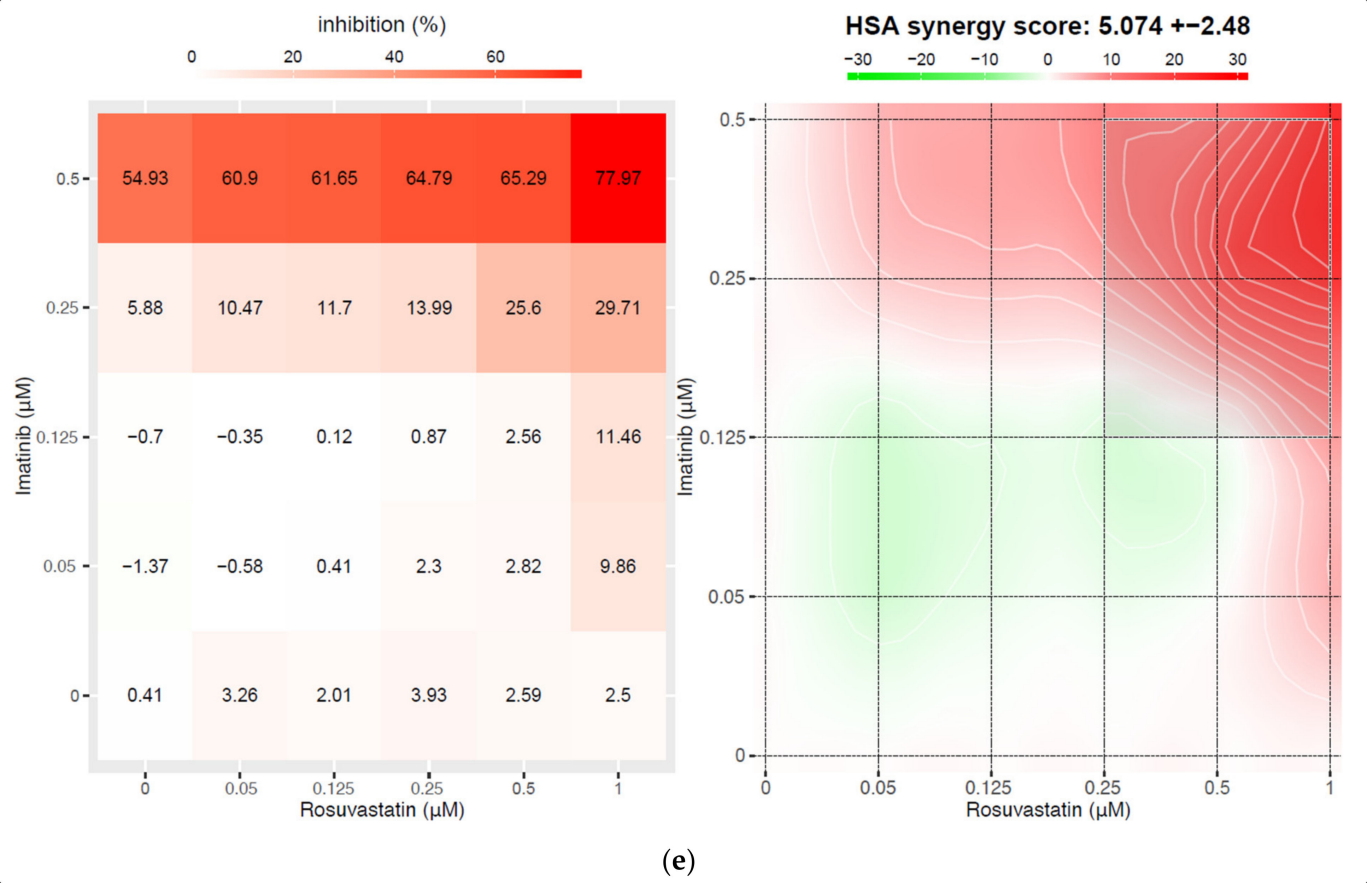
Effect of imatinib and rosuvastatin alone or in combination on the viability of K562/wildtype (WT), K562/T315I^mut^, and BaF3/T315I^mut^ cells. Viability of (**a**) K562/WT, (**b**) BaF3/T315I^mut^, and (**c**) K562/T315I^mut^ cells in different treatment groups. Results are presented as the mean±standard deviation of optical density (OD) values (*Y*-axis) from at least three independent measurements. The cell viability in the untreated control, rosuvastatin-treated, imatinib-treated, nilotinib-treated, dasatinib-treated, rosuvastatin/imatinib-treated, rosuvastatin/dasatinib-treated, and rosuvastatin/nilotinib-treated groups was examined at 72 h. (**d**) Phospho-CrkL/CrkL ratio assessed on the basis of *BCR-ABL1* activity in BaF3/T315I^mut^ cells treated with imatinib and/or rosuvastatin. The Phospho-CrkL/CrkL ratio relative to that in the non-treated control is presented as the mean ± standard deviation from at least three independent measurements determined using the colorimetric cell-based assay at 48 h. (**e**) Heatmap and synergy plot of K562 WT cells after rosuvastatin/imatinib treatment. On the heatmap (left), % cell death is represented by color gradient from low to high. On the synergy plot (right), combination scores are represented by color gradient from green (antagonism) to red (strong synergy). Data were analyzed using Student’s *t*-test with equal variance. *** *p* < 0.001, * *p* <0.05.

To investigate the cellular mechanisms underlying the increased molecular response of the statin/TKI in patients with CML undergoing imatinib therapy, the effects of statins (rosuvastatin and atorvastatin), TKIs (IM, NI, or DA), or various combinations and concentrations of TKIs and statins on the viability of K562 *BCR-ABL1*^+^ cells were examined. Cell viability in the treatment groups was compared to that of the control group. Rosuvastatin (1.5 μM) did not decrease the viability of K562/WT cells at 72 h post-treatment (80.52 ± 8.14% relative to that in the control group; *p* = 0.3027) (Figure 2a). However, the cell viability in the group treated with the rosuvastatin and IM (0.6 μM) combination was 17.90 ± 0.71%, relative to that in the control group (*p* < 0.0001) (Figure S2).

Consistently, the combination of rosuvastatin and NI or DA exerted additive growth-inhibitory effects against K562/WT cells. The cell viability in the group treated with the rosuvastatin and NI combination was 8.87 ± 1.77% relative to that in the control group (*p* < 0.001). Relative to that in the rosuvastatin and DA single treatment groups, the viability of cells was 3.73 ± 0.68% in the rosuvastatin and DA combination treatment groups (*p* < 0.001). Thus, statins enhanced the growth-inhibitory effects of TKIs against K562/WT cells (Figure S2).

As expected, the viability of BaF3/T315I*^mut^* cells, which is a TKI-resistant *BCR-ABL* mutant cell line, did not decrease upon treatment with IM, NI, or DA. However, the combination of rosuvastatin and TKIs exerted enhanced cytotoxic effects against BaF3/T315I*^mut^* cells (Figure 2b). Similar results were obtained with BaF3/G250E*^mut^* and BaF3/F317L*^mut^* cells (Figure S3).

Additionally, statins and TKIs exerted additive growth-inhibitory effects against K562/T315I*^mut^* cells, which were generated using CRISPR/Cas9-mediated gene editing. Treatment with IM did not significantly decrease the viability of K562/T315I*^mut^* cells, while the cell viability in the rosuvastatin and IM combination treatment group was reduced to 59.47 ± 3.39% relative to that in the control group (*p <* 0.001; Figure 2c). The results of the CrkL assay established that the combination of rosuvastatin and IM decreased K562/T315I*^mut^* cell viability by downregulating *BCR-ABL1* activity (Figure 2d). These findings indicate that the growth-inhibitory effect of the rosuvastatin/TKI combination against K562/T315I*^mut^* cells was significantly higher than that of rosuvastatin (*p* < 0.001) or TKI alone (*p* < 0.001). Thus, the combination of statins and TKIs could overcome ABL1 kinase domain mutation-mediated TKI resistance, including T315I mutation-mediated resistance.

To investigate whether the combined effects of statins and TKIs on CML cells were synergistic, the highest single agent (HSA) synergy score was calculated for each combination of imatinib and rosuvastatin in the K562 WT cell line. The combination of imatinib and rosuvastatin exerted a synergistic effect, with an HSA synergy score of 5.074 ± 2.48 (Figure 2e). The strongest inhibition was a combination of 0.5 μM imatinib and 1 μM statin (77.97%), and the strongest synergy was identified for the combination of 0.25 μM imatinib and 1 μM statin (HSA synergy score of 23.96).

### 3.3. Statins Suppress the Colony-Forming Capacity of Murine CML-KLS^+^ Cells In Vitro

**Figure 3:**
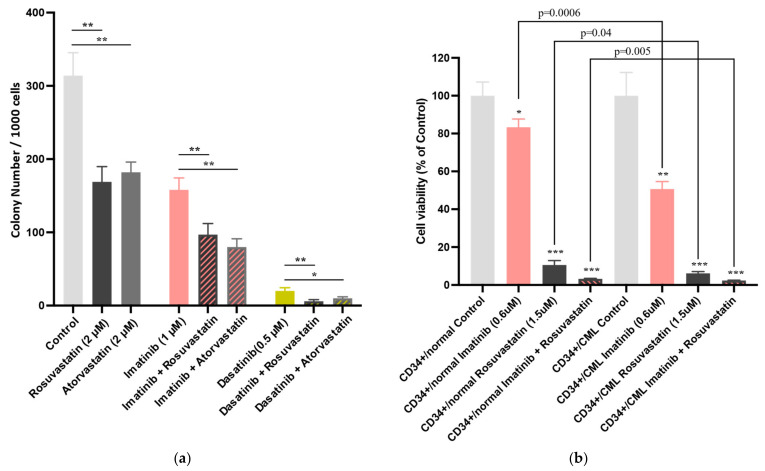
Effect of statins on murine chronic myeloid leukemia (CML)-KLS cells and human-derived cells in vitro. (**a**) Statins suppress the colony-forming capacity of murine CML-KLS cells in vitro. *cKit*+*Lineage*-*Sca1*+ cells isolated from tetracycline-inducible CML mice and Scl/Tal1-tTA/*tetO-BCR-ABL1* double transgenic mice were treated with tyrosine kinase inhibitors (1 µM imatinib/0.5 µM dasatinib) and statins (2 µM rosuvastatin/2 µM atorvastatin) for 3 days. (**b**) The bar plot shows the effect of the rosuvastatin (1.5 µM)/imatinib (0.6 µM) combination on human CD34^+^ cells isolated from clinical samples of patients with CML (CD34^+^/CML) and healthy individuals (CD34^+^/normal). Cell viability in the treatment group relative to that in the control group (*Y*-axis) at 192 h is represented as the mean± standard deviation from at least three independent measurements. Cell viability (%) was calculated as follows: (absorbance of the treatment group − absorbance of the blank group)/(absorbance of the control group − absorbance of the blank group). Data were analyzed using Student’s *t*-test with equal variance. The asterisk indicates significance, which was analyzed by comparing the control group’s results with those of the CD34^+^/normal or CD34^+^/CML group. *** *p* < 0.001, ** *p* < 0.01, * *p* < 0.05.

Next, we examined the effects of statins on the colony-forming capacity of freshly isolated CML-KLS^+^ cells in vitro. The CML-KLS^+^ cell/OP-9 stromal cell co-culture was treated with TKIs (IM (1 µM)/DA (0.5 µM)) and statins (rosuvastatin (2 µM)/atorvastatin (2 µM)) for 3 days. As shown in Figure 3a, the combination treatment significantly decreased the colony-forming capacity of murine CML-KLS^+^ cells in vitro. The colony-formation capacity of cells in the IM and rosuvastatin or atorvastatin combination treatment groups was 61.05 ± 9.48% (*p <* 0.01) or 50.53 ± 7.12% (*p <* 0.01), respectively, when compared with that in the control group. Additionally, the colony-formation capacity of cells in the DA and rosuvastatin or atorvastatin combination treatment groups was 32.48 ± 10.68% (*p <* 0.01) or 52.14 ± 10.68% (*p <* 0.05), respectively.

### 3.4. Combination of Rosuvastatin and IM Exert Growth-Inhibitory Effects Against CML CD34^+^ Cells

The in vitro effects of statins were examined in primary CD34+ leukemic cell fractions isolated from patients with blast crisis CML. CD34^+^ cells were isolated from two patients with CML (CD34^+^/CML) and one healthy control (CD34^+^/Norm) (Figure 3b). Next, these cells were treated with rosuvastatin and IM alone or in combination in vitro. The proliferation of untreated CD34^+^/CML cells was significantly higher than that of CD34^+^/Norm. CD34^+^/CML cells exhibited significantly lower viability than CD34^+^/Norm cells after treatment with IM (*p* = 0.0006) or rosuvastatin (*p* = 0.04). However, the viability of CD34^+^/CML cells in the rosuvastatin and IM combination treatment group was significantly lower than that in the IM (*p* < 0.01) and rosuvastatin single treatment groups (*p* < 0.001). The statin/IM combination exerted greater growth-inhibitory effects against CD34^+^/CML cells than against CD34^+^/Norm cells (*p* = 0.005). Thus, we concluded that a combination of rosuvastatin and IM exerted growth-inhibitory effects against CML CD34^+^ cells but not against normal CD34^+^ cells.

### 3.5. Statins Target the c-Myc and Hematopoietic Stem Cell Differentiation Pathways in CML

To examine the molecular mechanisms underlying the growth-inhibitory effects of the statin/TKI combination against CML cells, we performed a whole transcriptomic analysis. In total, 6243 DEGs were identified on the basis of the posterior probability of differential expression between the two groups. The log_2_ fold change values ranged from −6.89 to +3.24. The threshold value for the identification of DEGs was a 1.3-fold change. In total, 482 and 125 genes were downregulated and upregulated, respectively, in the rosuvastatin treatment group (Table S2).

**Figure 4:**
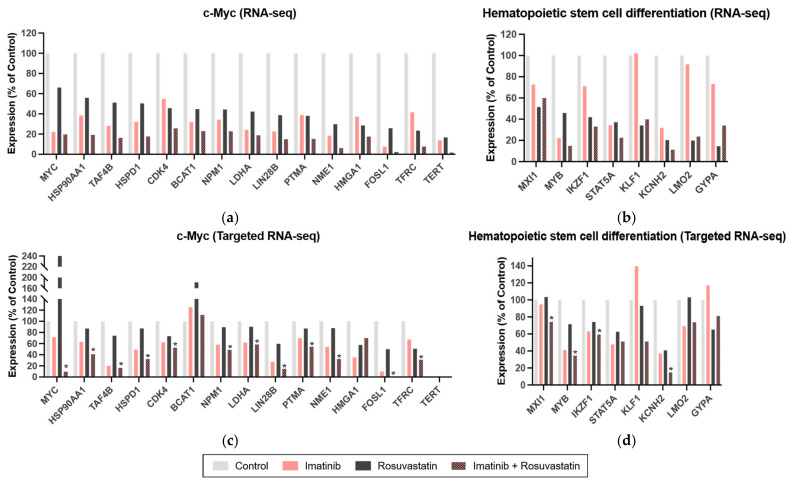
RNA sequencing analysis reveals that the combination of a statin and tyrosine kinase inhibitor downregulates the c-Myc and hematopoietic stem cell differentiation pathways. Expression of c-Myc (**a**) and hematopoietic stem cell differentiation (**b**) pathway-related genes as determined using RNA sequencing. Expression of genes related to the c-Myc pathway (**c**) and hematopoietic stem cell differentiation (**d**) pathway as determined using targeted RNA sequencing. Genes validated with targeted RNA sequencing are marked with an asterisk.

Pathway enrichment analysis using DAVID revealed that the gene set was significantly enriched in c-Myc (Figure 4a) and hematopoietic stem cell differentiation pathways (Figure 4b; false discovery rate < 0.05 for both) (Table S3). The combination of statins and TKIs suppressed the expression of genes in both pathways (Table S4). The results of the targeted RNA-seq assay were successfully replicated (Figure 4c,d).

## 4. Discussion

The findings of this study suggest that statins can be repurposed for improving the efficacy of TKI therapy against CML. Clinical data suggested that the concomitant use of statins improved DMR rates in patients with CML undergoing IM therapy (55.8% vs. 41.0%; DMR rates at 5 years in patients who received concurrent statin therapy vs. those not receiving statin therapy; *p* = 0.001). This difference may not be directly related to statin effects; however, it can result from other confounding factors directly or indirectly linked to the use of statins. For example, the patients in the group receiving statins were older and consumed a higher number of other concurrent medications that could potentiate drug interactions with TKIs compared with those in the group not receiving statins. To exclude the interaction with these confounding factors, a PSM method was applied to balance out other confounding factors, such as age. PSM analysis after controlling for age revealed that the DMR rates in the statin group were higher than those in the non-statin group.

The results of the in vitro studies revealed that the combination of statin and TKI exerted additive cytotoxic effects against human CML cells and mouse BaF3 cells (including those harboring *ABL1* kinase domain mutations, such as the T315I mutation). Additionally, the combination of statins and TKIs exerted enhanced cytotoxic effects against murine CML-KLS^+^ cells, indicating that statins can potentially inhibit/eradicate leukemic progenitor cells in patients with CML. Furthermore, the RNA-seq data revealed that the statin/TKI combination downregulated the c-Myc and hematopoietic stem cell differentiation pathways. Thus, these pathways are potential therapeutic targets for the eradication of leukemic progenitor cells in patients with CML.

Quiescent leukemic stem cells are often resistant to both conventional chemotherapy and targeted therapies and are retained after the discontinuation of therapy, contributing to relapse [26]. Thus, it is important to isolate a stem cell compartment and entrance and exit from the quiescent state of leukemic stem cells.

The mechanism of resistance of CML stem cells has been extensively investigated. Several pathways, including the JAK-STAT [27,28,29], Hedgehog [30,31,32], β-catenin [33,34,35,36], and PI3K [37,38,39] pathways, have been reported to be involved in the therapy resistance of CML stem cells. One study demonstrated that *c-Myc* and *TP53* mediated the survival network in CML stem cells [40]. Targeting *c-Myc* and/or *TP53* is an ideal therapeutic strategy for eradicating leukemic progenitor cells in CML. However, inhibitors of the c-Myc pathway have not been successfully identified.

This study hypothesized that the *c-Myc*-mediated pathway is a potential target of statins in the presence or absence of TKIs. The results from some studies have suggested that statins regulate the *c-Myc*-mediated pathway. Statin-regulated microRNAs repress human *c-Myc* expression and function [41]. HMGCR, which is reported to regulate c-Myc phosphorylation and activation, enhances the tumorigenic potential of hepatocellular carcinoma [42]. The RNA-seq data in this study support the hypothesis that statins inhibit the c-Myc pathway in CML cells, which further demonstrated that *c-Myc* is a target of statins. Thus, the additive growth-inhibitory activity of TKIs and statins against CML cells may be mediated through the blockade of the c-Myc pathway.

Another potential confounder is the statin subtype, which can affect drug interactions with TKI drugs. Atorvastatin and simvastatin, but not rosuvastatin and fluvastatin, are metabolized by *CYP3A4/3A5* [43,44]. Thus, two different types of statins (rosuvastatin and atorvastatin) were analyzed (Figure 3). The growth-inhibitory activities of rosuvastatin and atorvastatin against murine CML-KLS^+^ cells were not significantly different. In addition, we did not observe any difference in response to imatinib therapy according to the statin subtype in our clinical outcomes (data not shown).

Statins can also enhance the cytotoxic effects of TKIs by inhibiting a transmembrane pump, which can potentiate the intracellular concentrations of TKIs. Glodkowska-Mrowka et al. suggested that statins increased the intracellular concentrations of IM in primary CML cells and cell lines through the inhibition of the membrane efflux transporters ABCB1 and ABCG2 [45]. However, RNA-seq data analysis in this study revealed that statins did not affect the expression levels of *ABCB1*/*ABCG2* in K562 cells (data not shown). The enhanced intracellular concentration of TKIs through the statin-mediated inhibition of the ABCB1/ABCG2 drug transporter activity cannot explain the additive growth-inhibitory effects of statins against BaF3/T315I*^mut^* or K562/T315I*^mut^* cells, which are highly resistant to increased concentrations of TKIs [46]. Thus, the combination of statins may block alternative pathways to inhibit CML cell growth, such as the c-Myc pathway.

Although ponatinib or asciminib are suggested to be effective against T315I mutant CML cells, a higher dose of treatment is recommended for maximizing the chance of achieving a molecular response [47,48]. However, if the combination of statin and TKI is effective against T315I mutant CMLs, it can serve as a feasible approach for adding a statin in the treatment of CML patients with T315I mutation. In addition, the quality of life (QoL) of patients is a considerable issue in TKI therapy. Our study indicates that the use of statins improved treatment outcomes in CML patients when combined with IM therapy; however, we did not confirm this observation with next-generation TKI therapy. Patients treated with dasatinib reported better disease-specific health-related QoL outcomes than those treated with imatinib [49]. Therefore, it would be clinically useful to evaluate the effect of statins on the QoL of patients treated with second-generation TKIs if it could further improve QoL.

Additionally, in a study on chronic neutrophilic leukemia (CNL) and atypical (BCR-ABL1–negative) CML in patients with *CSF3R* mutations, *CSF3R* truncating mutations were found to be sensitive to dasatinib [50] suggesting that the trial of SRC kinase inhibitors is a reasonable approach [51]. On the basis of the results that the growth-inhibitory effects of statins in combination with dasatinib against CML cells were *BCR-ABL* mutation-independent, statins may also benefit the treatment of these diseases.

Approximately 50–60% of patients with CML achieve a stable DMR after TKI therapy [52,53]. TKI therapy can be successfully discontinued in these patients. The achievement of DMR is a prerequisite for treatment-free remission (TFR). Hence, various compounds have been investigated as potential candidates for enhancing the molecular response during TKI therapy [54,55,56]. Compared with the development of several potential but experimental compounds, repurposing statins is a more straightforward solution for enhancing the molecular response in CML treatment. Second-generation TKIs have been reported to exhibit cardiovascular toxicity. Thus, statins have been widely prescribed for patients with CML undergoing second-generation TKI therapy for modification of their cardiovascular risk factors, such as hyperlipidemia [57]. Furthermore, the results of this study support the therapeutic benefit of the concomitant use of statins in TKI therapy for patients with CML.

## 5. Conclusions

Our results suggest that the combination of statins and TKIs can augment the eradication of CML progenitor cells in in vitro models. Additionally, the additive effects of statins and TKIs enhance the DMR rate in patients with CML. The potential additive effects of statins and TKIs are clinically relevant for patients with CML. In particular, this combination is a strong candidate for achieving DMR in patients with CML who have not achieved DMR with TKI therapy alone. For these patients, combining statins with TKI therapy may promote the achievement of DMR and subsequently enable TKI discontinuation for TFR.

## Figures and Tables

**Table 1:** Demographic and disease characteristics of patients at the time of imatinib therapy initiation.

Patient Characteristics	Overall	Number of Patients (*n* = 408)		*p*-Value	Selected 84 Case-Control Pairs for PSM		*p*-Value
		Statin Group	Non-Statin Group		Statin Group	Non-Statin Group	
Number of patients; *n* (%)	408	88 (21.3)	320 (78.7)	-	84 (50.0)	84 (50.0)	-
Age (years); median (range)	52 (17–83)	62 (24–83)	49 (17–82)	<0.001	62 (24–77)	62 (22–78)	0.813
Gender (female:male); *n* (%)	175/231	39/49	136/182	0.795	39/45 (46.4:53.6)	44/40 (52.4:47.6)	0.44
	(43.1:56.9)	(44.3:55.7)	(42.8:57.2)				
Previous treatment; *n* (%)	115 (28.2)	29 (33.0)	86 (26.9)	0.262	28 (33.3)	32 (38.1)	0.52
Previous history of interferon therapy; *n* (%)	105 (25.7)	26 (29.5)	79 (24.7)	0.356	26 (31.0)	29 (34.5)	0.622
Previous history of BMT; *n* (%)	16 (3.9)	4 (4.5)	12 (3.8)	0.757	3 (3.6)	4 (4.8)	0.699
Sokal risk group; *n* (%)	170 (41.7)	34 (38.6)	136 (42.5)	0.785	33 (39.3)	30 (35.7)	0.888
	120 (29.4)	28 (31.8)	92 (28.8)		25 (29.8)	26 (31.0)	
	118 (28.9)	26 (29.5)	92 (28.8)		26 (31.0)	28 (33.3)	
Additional chromosomal abnormalities *; *n* (%)	25 (6.1)	5 (5.7)	20 (6.3)	0.844	4 (4.8)	2 (2.4)	0.682
**Response**							
Median follow-up (months)	72	69	69	0.376	74	75	0.705
MCyR at 6 months (% ± SE)	68.9 ± 4.7	76.2 ± 9.5	68.5 ± 5.4	0.38	76.2 ± 9.7	72.0 ± 10.1	0.684
CCyR at 12 months (% ± SE)	64.4 ± 4.9	70.1 ± 10.3	62.8 ± 5.6	0.091	71.1 ± 10.4	67.1 ± 10.5	0.417
MMR at 18 months (% ± SE)	53.1 ± 5.2	67.3 ± 10.5	49.2 ± 5.8	0.005	68.2 ± 10.7	53.1 ± 11.1	0.072
DMR at 5 years (% ± SE)	44.2 ± 5.5	55.8 ± 11.7	41.0 ± 6.1	0.001	56.8 ± 11.9	47.0 ± 11.6	0.019
FTF at 3 years (% ± SE)	87.6 ± 1.7	90.2 ± 3.3	86.9 ± 2.0	0.525	89.8 ± 3.4	91.9 ± 3.7	0.953
PFS at 5 years (% ± SE)	95.4 ± 1.1	96.5 ± 2.0	95.0 ± 1.4	0.686	96.4 ± 2.0	97.3 ± 1.9	0.938
OS at 5 years (% ± SE)	97.4 ± 0.1	98.8 ± 1.2	97.0 ± 0.1	0.542	98.8 ± 1.2	97.3 ± 1.9	0.734

PSM, propensity score matching; BMT, bone marrow transplantation; MCyR, major cytogenetic response; CCyR, complete cytogenetic response; MMR, major molecular response; DMR, deep molecular response; FTF, freedom from treatment failure; PFS, progression-free survival; OS, overall survival; SE, standard error. *-Y (*n* = 5); double Ph (*n* = 3); t(9;22;22) (*n* = 2); -X (*n* = 1); inv(5) (*n* = 1); inv(9q) (*n* = 1); inv(11) (*n* = 1); t(1;22;8), t(1;22), der(1), del(9), der(22), der(9) (*n* = 1); t(2;9;22) (*n* = 1); t(5;9;22) (*n* = 1); t(6;9;22) (*n* = 1); t(7;8) (*n* = 1); t(7;9;22) (*n* = 1); t(8;16) (*n* = 1); t(9;17),t(15;22),t(17;22) (*n* = 1); t(9;17;22) (*n* = 1); t(15;19)(*n* = 1); +8 (*n* = 1);

## Data Availability

The data presented in this study are available on request from the corresponding author.

## Supplementary Materials

The following are available online at https://www.mdpi.com/article/10.3390/cancers13215543/s1, Figure S1: Overall study design and workflow, Figure S2: Effect of statins and/or tyrosine kinase inhibitors (TKIs) on K562 cell viability, Figure S3: Growth-inhibitory effects of the combination of rosuvastatin and tyrosine kinase inhibitors against various BaF3/mutant cells, Table S1: Drug administration, Table S2: List of downregulated and upregulated genes in rosuvastatin-treated cells determined using RNA sequencing, Table S3: Pathway enrichment analysis of differentially expressed genes between the control and rosuvastatin-treated groups, Table S4: List of candidate genes that overlap with those determined in the pathway enrichment analysis using DAVID.
