# Supplementary material for: Statins Enhance the Molecular Response in Chronic Myeloid Leukemia when Combined with Tyrosine Kinase Inhibitors
Source: Cancers (Basel). 2021 Nov 4;13(21):5543. doi: 10.3390/cancers13215543 (PMC8582667; doi:10.3390/cancers13215543)
Supplement: Supplementary file 1 [file cancers-13-05543-s001.zip › cancers-1414870-supplementary.pdf]

SUPPLEMENTARY MATERIALS

1. Supplemental Methods

1.1. Evaluation of deep molecular response to tyrosine kinase inhibitor (TKI) therapy according to the concomitant use of statin in patients with CML

A total of 408 patients who met the inclusion criteria were included in the analysis. In particular, this study focused on patients diagnosed with chronic phase CML who were treated with IM (400 mg/day) as frontline therapy. The response to TKI therapy was evaluated on the basis of complete cytogenetic response (CCyR), major molecular response (MMR), molecular response at 4.5 ($MR^{4.5}$), and statin use at the Princess Margaret Cancer Center (Toronto, Canada). The statin group was defined as patients who had been prescribed a statin for hypercholesterolemia at the time of IM therapy initiation and who were treated with statins for at least 3 years along with IM therapy.

The demographic and disease characteristics of patients at the time of initiation of IM therapy are presented in Table 2. The levels of *BCR-ABL1* transcripts in peripheral blood samples were examined using quantitative real-time polymerase chain reaction analysis once every three months. The *BCR-ABL1* transcript levels were measured and reported using the International Scale, according to the international recommendations established for the standardization of this test.

The response criteria used in this study were similar to those previously defined in other studies. Briefly, cytogenetic responses were categorized as complete (CCyR; 0% Ph^+ cells in the bone marrow as determined using conventional cytogenetic techniques), partial (1% to 34% Ph^+ cells in the bone marrow), or minor (35% to 90% Ph^+ cells in the bone marrow). A major cytogenetic response was defined as the sum of CCyR and partial cytogenetic response (i.e., 0%–35% Ph^+ cells in the bone marrow). MMR was defined as a *BCR-ABL1* transcript level ≤0.1%^{IS}. Moreover, a molecular response of ≤4.5 log reduction ($MR^{4.5}$) was defined as a *BCR-ABL1* transcript level of 0.0032%^{IS}, equivalent to a 4.5-log reduction in the *BCR-ABL1* transcript level. Treatment failure was defined as events, including primary hematologic resistance, cytogenetic resistance, loss of CCyR, development of tyrosine kinase domain mutations, clonal evolution, and progression to accelerated phase (AP) or blast crisis (BC). Time to progression-free survival (PFS) was defined as the interval between initiation of IM therapy and progression to accelerated AP or BC or death from any cause. Overall survival (OS) was calculated from the initiation of IM therapy until the date of death from any cause or until the last follow-up. Additionally, other reasons for IM discontinuation were recorded.

The cumulative incidences of CCyR, MMR, and $MR^{4.5}$ were calculated considering competing risks (IM discontinuation or death). The probabilities of freedom from treatment failure (FTF) and PFS/OS were estimated using the Kaplan–Meier method.

The cumulative incidences of CCyR, MMR, and $MR^{4.5}$, were compared and plotted according to the use of statins using the Gray test. FTF, PFS, and OS were analyzed using the log-rank test. For multivariate analyses, the Fine–Gray methods were adopted for analyzing CCyR, MMR, and $MR^{4.5}$, and the Cox proportional hazard models for analyzing FTF, PFS, and OS. A stepwise selection algorithm was applied for model selection using the criteria for variable selection (p = 0.05) and variable removal

($p = 0.1$). The following variables were considered for modeling: statin use, age (continuous variable), gender, Sokal risk group (low/intermediate vs. high risk), and additional cytogenetic abnormalities in the Ph^{+} clone. Hazard ratios and 95% confidence intervals (CIs) were estimated for significant risk factors based on multivariate analysis. Differences were considered significant at $p < 0.05$.

1.2. Propensity score matching (PSM) analysis

PSM analysis was performed to exclude any effect of the potential interaction of confounding factors between the use of statins and other clinical factors that are potentially associated with the response rate to IM therapy. Pre-treatment variables included in the PSM analysis were age, sex, Sokal risk group, and additional cytogenetic abnormalities. A case-control study with well-balanced pairs of patients treated with or without statins was performed using PSM analysis. A total of 84 case-control pairs were selected for this study. The difference in the propensity scores between pairs was within 0.05. Paired analysis was adopted for PSM analysis of survival [3].

1.3. Statistical analysis

The cumulative incidence of response to IM therapy was calculated considering the competing risk of IM discontinuation or death. Gray's test was used for comparison. The Fine–Gray model was used for multivariate analysis. Student's *t*-test was used for analyzing the mean percentage viability of the different groups. All statistical analyses were performed using R (version 3.2.0; R Foundation for Statistical Computing, Austria) and the EZR software.

1.4. Determining the phospho-CrkL/CrkL ratio

To measure the pCrkL/CrkL ratio, the cells treated with imatinib (IM; Gleevec; Novartis, Switzerland) and/or rosuvastatin (Selleckchem, Houston, TX, USA) were assayed using the phospho-CrkL (Tyr207) colorimetric cell-based enzyme-linked immunosorbent assay kit (Aviva Systems Biology, San Diego, CA, USA). The optical density at 450 nm (OD_{450}) of phosphorylated CrkL was normalized to that of non-phosphorylated CrkL protein (OD_{450} anti-CrkL P-Tyr207 antibody)/OD_{450} anti-CrkL antibody). Next, the cells were stained with crystal violet and solubilized in sodium dodecyl sulfate. The OD_{595} value of the mixture was determined using an xMark microplate absorbance spectrophotometer (Bio-Rad Laboratories, Hercules, CA, USA). The OD_{450} values were normalized to the OD_{595} values (OD_{450}/OD_{595}).

1.5. Double transgenic (tg) murine model of chronic myeloid leukemia (CML)

Scl/Tal1-tTA (JAX strain 6209) and *tetO-BCR-ABL1* (JAX strain 6202) tg mice, which were purchased from the Jackson Laboratory (Bar Harbor, ME, USA), were interbred to generate Scl/Tal1-tTA/*tetO-BCR-ABL1* double tg mice, which were maintained in cages and provided drinking water containing 20 μg/mL doxycycline (Dox Sigma-Aldrich, St. Louis, MO, USA). Dox-supplemented drinking water was replaced with unsupplemented drinking water at week 5 post-birth. The

consumption of unsupplemented drinking water induces the expression of the *BCR-ABL1* oncogene. The CML-like disease developed in the double tg mice at approximately week 5 after Dox withdrawal.

1.6. Isolation of cKit⁺Lineage⁻Sca1⁺ (KLS) cells from the murine CML model

To evaluate the colony-forming capacity of murine CML-KLS⁺ cells co-cultured with OP-9 stromal cells *in vitro*, we isolated KLS cells from tetracycline-inducible (Tet-on) CML mice. Bone marrow cells were isolated from the two hind limbs of each Tet-on CML mouse at week 5 after Dox withdrawal. Next, the cells were stained with phycoerythrin-conjugated anti-Sca-1 (E13−161.7), fluorescein isothiocyanate (FITC)-conjugated anti-CD4 (L3T4), FITC-conjugated anti-CD8 (53-6.7), FITC-conjugated anti-B220 (RA3-6B2), FITC-conjugated anti-TER119 (Ly-76), FITC-conjugated anti-Gr-1 (RB6-8C5), FITC-conjugated anti-Mac1 (M1/70)(all from BD Biosciences Pharmingen, San Diego, CA, USA), and allophycocyanin-conjugated anti-c-Kit (ACK2) (eBioscience, San Diego, CA, USA) antibodies. The cells were subsequently sorted using a flow cytometer (BD FACSAria III; BD Biosciences) for isolation of the fractions containing KLS cells.

1.7. Colony-forming capacity of murine CML-KLS⁺ cells

Freshly isolated murine CML-KLS⁺ cells were co-cultured with OP-9 stromal cells under hypoxic conditions (3% O_2) at 37 °C in the presence of dimethyl sulfoxide (control), IM, dasatinib (DA; Sprycel; Selleckchem), and statins (rosuvastatin or atorvastatin; Selleckchem, Houston, TX, USA). The cells were harvested, washed with phosphate-buffered saline, and cultured in a semi-solid methylcellulose medium containing the following cytokines under hypoxic conditions: stem cell factor, interleukin (IL)-3, IL-6, and erythropoietin (MethoCult GF M3434; Stemcell Technologies, Canada). Colony formation was evaluated under a microscope after seven days. The data were analyzed using unpaired Student's *t*-test.

1.8. RNA sequencing (RNA-seq) analysis

The cells were incubated with rosuvastatin (2.0 μM) at 37 °C and 5% CO_2. Next, the cells were harvested 24 h after drug administration. Total RNA was extracted from each sample (control and statin-treated groups) using TRIzol reagent (Invitrogen, Waltham, MA, USA). Each sample was prepared in triplicate for differentially expressed gene (DEG) analysis. The RNA-seq library was prepared using the TruSeq RNA Sample Prep Kit v2 to capture the coding transcriptome without strand information. To prepare the sequencing library, total RNA was subjected to random fragmentation and reverse-transcribed to cDNA using random hexamer primers. Next, the cDNA was ligated with the 5 and 3 adapters. Non-stranded RNA-seq was performed on a HiSeq 2500 (Illumina, San Diego, CA, USA) with 2 × 101 bp paired-end sequencing.

1.9. Bioinformatics analysis

Adapter trimming was performed using Trimmomatic-0.33 before the sequencing reads were aligned with the reference genome (Ensembl, GRCh37). To remove Illumina-specific adapter sequences,

the trimming threshold was determined using ILUMINACLIP. A maximum of two mismatches was allowed with a clipping paired-end read score of 30. The adapter sequences (TruSeq3-PE adapter) were generated for TruSeq library construction. The trimmed paired reads were aligned with the human reference genome (release version GRCh37) with Ensembl General Transfer Format gene annotation using STAR 2.4.2a. Ensembl gene annotation files were downloaded from Ensembl (release 75) through Illumina iGenomes collection of reference sequences and annotation files. Ensembl release 75 comprised 20,805 coding genes, 9,096 short non-coding genes, 13,870 long non-coding genes, and 14,181 pseudogenes. RNA-seq data quality was evaluated using RSeQC v2.6.4. To calculate the expression level, we generated raw read counts using RSEM v1.3.0. The total number of reads within each sample was computed using the upper-quartile (UQ) normalization. DEGs between the control and statin-treated groups were analyzed using EBseq. Mean expression levels were calculated from triplicate samples from each group. Genes exhibiting low expression (average normalized UQ < 100) were excluded from the pathway enrichment analysis.

1.10. Targeted RNA-seq assay

Customized Precise™ assays with molecule-specific barcode molecular indexing were designed to simultaneously analyze 200 genes (BD Biosciences, San Jose, CA, USA) (Supplementary Table 5). K562 cells (treated with rosuvastatin in the presence or absence of IM or dimethyl sulfoxide) were seeded into a Precise™ 96-well assay plate (BD Biosciences) (500 cells/well). We subsequently processed the samples as per the Precise™ assay protocol to generate a sequencing library. The amplified library was sequenced on a HiSeq 2500 platform (Illumina). Data deconvolution was performed with an automated algorithm using a Seven Bridges Genomics pipeline (tailor-made for BD Precise generated datasets).

1.11. Pathway enrichment analysis

Gene molecular function and/or biological processes of the identified gene classes were analyzed using both gene ontology enrichment in ConsensusPathDB [1] and DAVID [2]. Enriched pathway-based sets were presented using over-representation analysis, computed on the basis of the contained count in the predefined gene sets from the input candidate gene list. The p-value, calculated according to the hypergeometric test based on the number of overlapping genes, was corrected for multiple testing using the false discovery rate method.

References

1. Kamburov, A.; Wierling, C.; Lehrach, H.; Herwig, R. ConsensusPathDB--a database for integrating human functional interaction networks. *Nucleic Acids Res* **2009**, *37*, D623-628, doi:10.1093/nar/gkn698.
2. Huang da, W.; Sherman, B.T.; Lempicki, R.A. Bioinformatics enrichment tools: paths toward the comprehensive functional analysis of large gene lists. *Nucleic Acids Res* **2009**, *37*, 1-13, doi:10.1093/nar/gkn923.
3. Kanda, Y. Investigation of the freely available easy-to-use software 'EZR' for medical statistics. *Bone Marrow Transplant* **2013**, *48*, 452-458, doi:10.1038/bmt.2012.244.

2. Supplementary Figure and Table List

Figure S1	Overall study design and workflow.
Figure S2	Effect of statins and/or tyrosine kinase inhibitors (TKIs) on K562 cell viability.
Figure S3	Growth-inhibitory effects of the combination of rosuvastatin and tyrosine kinase inhibitors against various BaF3/mutant cells.
Table S1	Drug administration.
Table S2	List of downregulated and upregulated genes in rosuvastatin-treated cells, as determined using RNA sequencing.
Table S3	Pathway enrichment analysis of differentially expressed genes between the control and rosuvastatin-treated groups.
Table S4	List of candidate genes that overlap with those determined in the pathway enrichment analysis using DAVID.

Figure S1. Overall study design and workflow.

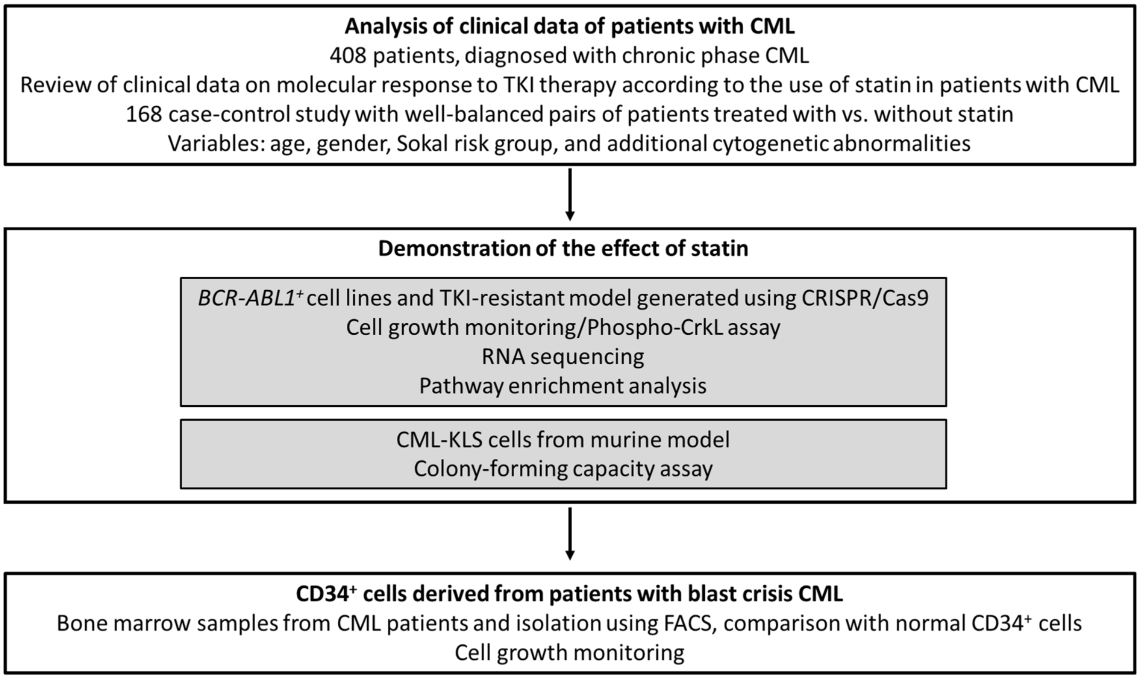

Figure S2. Effect of statins and/or tyrosine kinase inhibitors (TKIs) on K562 cell viability. Percentage viability of K562 cells treated with statins, TKIs, or a combination of statin and imatinib at 72 h post-treatment. Data are presented as the mean ± standard deviation from at least two independent measurements. Data were analyzed using the Student's *t*-test with equal variance. *** $p < 0.001$, ** $p < 0.01$.

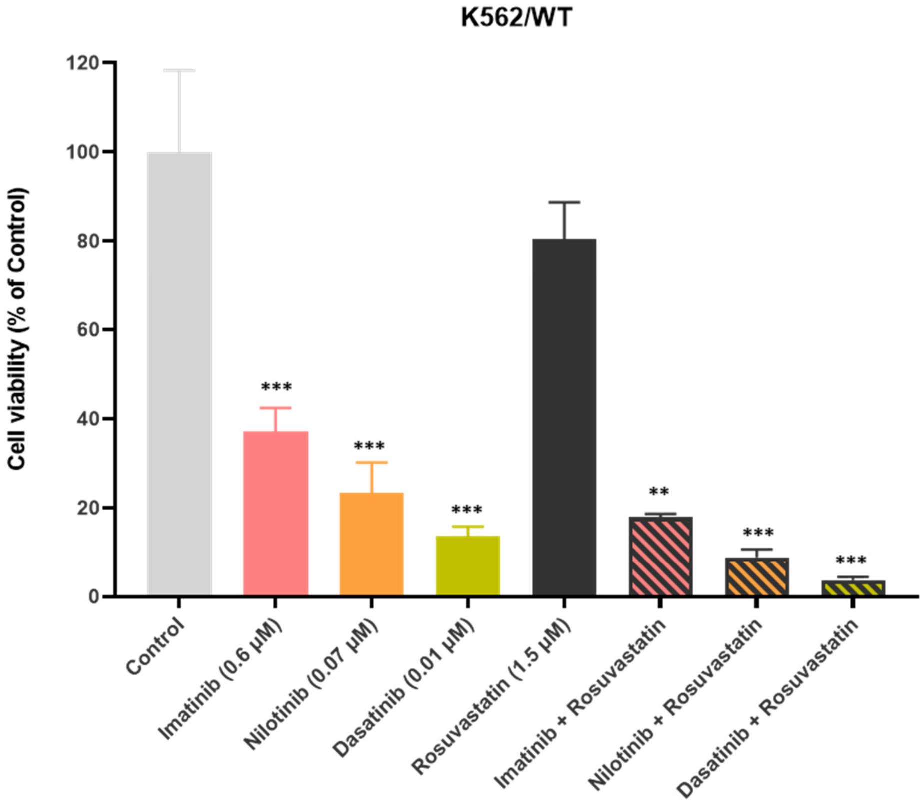

Figure S3. Growth-inhibitory effects of the combination of rosuvastatin and tyrosine kinase inhibitors against various BaF3/mutant cells. Viability of (**a**) BaF3/G250E^{mut} and (**b**) BaF3/F317L^{mut} cells in different treatment groups. Data are presented as the mean ± standard deviation of optical density (OD) value (Y-axis) from at least three independent measurements. Viability of cells in the untreated control, rosuvastatin-treated, imatinib-treated, nilotinib-treated, rosuvastatin/imatinib-treated, and rosuvastatin/nilotinib-treated groups were determined at 72 h post-treatment. The data were analyzed using Student's *t*-test with equal variance. *** $p < 0.001$, ** $p < 0.01$.

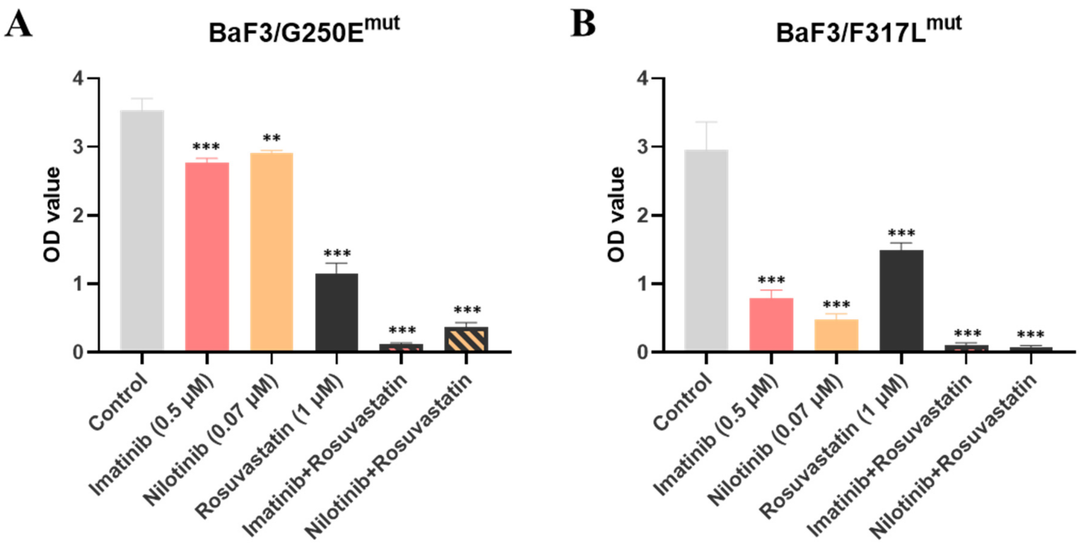

(**a**) (**b**)

Table S1. Drug administration.

	Imatinib	Nilotinib	Dasatinib	Rosuvastatin	Atorvastatin
**Wild-type mouse HSCs**	2 μM	-	-	2 μM	2 μM
**CML mouse-derived KLS cells**	1 μM	-	0.5 μM	2 μM	2 μM
**Human CML-derived cell line**	0.6 μM	0.07 μM	0.01 μM	2 μM	-
**Mouse leukemia-like cell line (Ba/F3 cells)**	0.5 μM	0.07 μM	0.01 μM	1 μM	-

HSC, hematopoietic stem cells; CML, chronic myeloid leukemia; KLS cells, *cKit*$^{+}$*Lineage*$^{-}$*Sca1*$^{+}$ cells

Table S2. List of downregulated and upregulated genes in rosuvastatin-treated cells determined using RNA sequencing.

Gene (Ensembl ID_GeneSymbol)	PPEE	PPDE	Log_2 fold change
ENSG00000258017_RP11-386G11.10	0	1	−6.89
ENSG00000225107_AC092484.1	0	1	−4.03
ENSG00000237311_RP6-159A1.3	0	1	−3.92
ENSG00000135218_CD36	0	1	−3.67
ENSG00000136929_HEMGN	0	1	−3.48
ENSG00000125740_FOSB	0	1	−3.40
ENSG00000130656_HBZ	0	1	−3.21
ENSG00000228742_RP5-884M6.1	0	1	−3.13
ENSG00000229988_HBBP1	0	1	−3.01
ENSG00000163285_GABRG1	0	1	−2.92
ENSG00000110079_MS4A4A	0	1	−2.88
ENSG00000223609_HBD	0	1	−2.86
ENSG00000104368_PLAT	0	1	−2.84
ENSG00000127533_F2RL3	0	1	−2.80
ENSG00000130294_KIF1A	0	1	−2.73
ENSG00000188643_S100A16	0	1	−2.70
ENSG00000229140_CCDC26	0	1	−2.68
ENSG00000182459_TEX19	0	1	−2.62
ENSG00000143627_PKLR	0	1	−2.62
ENSG00000175592_FOSL1	0	1	−2.61
ENSG00000075035_WSCD2	0	1	−2.57
ENSG00000135363_LMO2	0	1	−2.55
ENSG00000129951_LPPR3	0	1	−2.51
ENSG00000115461_IGFBP5	0	1	−2.48
ENSG00000151276_MAGI1	0	1	−2.46
ENSG00000237289_CKMT1B	0	1	−2.46
ENSG00000081237_PTPRC	0	1	−2.42
ENSG00000017483_SLC38A5	0	1	−2.42
ENSG00000069482_GAL	0	1	−2.42
ENSG00000223572_CKMT1A	0	1	−2.40
ENSG00000196154_S100A4	0	1	−2.38
ENSG00000178821_TMEM52	0	1	−2.37
ENSG00000260923_AC137934.1	0	1	−2.36
ENSG00000100234_TIMP3	0	1	−2.36
ENSG00000075340_ADD2	0	1	−2.35
ENSG00000118503_TNFAIP3	0	1	−2.34
ENSG00000188707_ZBED6CL	0	1	−2.32

ENSG00000147465_STAR	0	1	−2.31
ENSG00000099260_PALMD	0	1	−2.25
ENSG00000188536_HBA2	0	1	−2.24
ENSG00000135046_ANXA1	0	1	−2.23
ENSG00000165646_SLC18A2	0	1	−2.22
ENSG00000119865_CNRIP1	0	1	−2.22
ENSG00000267243_AC005307.3	0	1	−2.21
ENSG00000086506_HBQ1	0	1	−2.19
ENSG00000165810_BTNL9	0	1	−2.19
ENSG00000116678_LEPR	0	1	−2.17
ENSG00000115641_FHL2	0	1	−2.17
ENSG00000236194_AC003104.1	0	1	−2.17
ENSG00000163554_SPTA1	0	1	−2.15
ENSG00000175449_RFESD	0	1	−2.14
ENSG00000265843_LINC01029	0	1	−2.14
ENSG00000110092_CCND1	0	1	−2.13
ENSG00000187091_PLCD1	0	1	−2.12
ENSG00000134369_NAV1	0	1	−2.12
ENSG00000254979_RP11-872D17.8	0	1	−2.11
ENSG00000119888_EPCAM	0	1	−2.11
ENSG00000127561_SYNGR3	0	1	−2.10
ENSG00000173621_LRFN4	0	1	−2.09
ENSG00000196565_HBG2	0	1	−2.09
ENSG00000225528_RP3-370M22.8	0	1	−2.08
ENSG00000135821_GLUL	0	1	−2.08
ENSG00000226887_ERVMER34-1	0	1	−2.08
ENSG00000120738_EGR1	0	1	−2.05
ENSG00000249859_PVT1	0	1	−2.05
ENSG00000182272_B4GALNT4	0	1	−2.05
ENSG00000102010_BMX	0	1	−2.04
ENSG00000172927_MYEOV	0	1	−2.04
ENSG00000206172_HBA1	0	1	−2.04
ENSG00000198336_MYL4	0	1	−2.02
ENSG00000213931_HBE1	0	1	−2.02
ENSG00000178773_CPNE7	0	1	−2.01
ENSG00000213934_HBG1	0	1	−2.00
ENSG00000167286_CD3D	0	1	−2.00
ENSG00000263585_RP11-498C9.13	0	1	−1.99
ENSG00000170180_GYPA	0	1	−1.99
ENSG00000007264_MATK	0	1	−1.99
ENSG00000128283_CDC42EP1	0	1	−1.99

ENSG00000153303_FRMD1	0	1	−1.98
ENSG00000135655_USP15	0	1	−1.98
ENSG00000242180_OR51B5	0	1	−1.98
ENSG00000198915_RASGEF1A	0	1	−1.98
ENSG00000180644_PRF1	0	1	−1.97
ENSG00000175084_DES	0	1	−1.97
ENSG00000182368_FAM27A	0	1	−1.96
ENSG00000167355_AC104389.28	0	1	−1.95
ENSG00000184363_PKP3	0	1	−1.95
ENSG00000169247_SH3TC2	0	1	−1.95
ENSG00000198756_COLGALT2	0	1	−1.94
ENSG00000153721_CNKSR3	0	1	−1.93
ENSG00000182752_PAPPA	0	1	−1.93
ENSG00000169442_CD52	0	1	−1.92
ENSG00000151632_AKR1C2	0	1	−1.92
ENSG00000104267_CA2	0	1	−1.92
ENSG00000141744_PNMT	0	1	−1.91
ENSG00000189337_KAZN	0	1	−1.91
ENSG00000173898_SPTBN2	0	1	−1.91
ENSG00000137648_TMPRSS4	0	1	−1.89
ENSG00000100285_NEFH	0	1	−1.89
ENSG00000055118_KCNH2	0	1	−1.89
ENSG00000157303_SUSD3	0	1	−1.88
ENSG00000162511_LAPTM5	0	1	−1.88
ENSG00000112715_VEGFA	0	1	−1.88
ENSG00000109534_GAR1	0	1	−1.88
ENSG00000149557_FEZ1	0	1	−1.88
ENSG00000100300_TSPO	0	1	−1.87
ENSG00000151689_INPP1	0	1	−1.87
ENSG00000169174_PCSK9	0	1	−1.87
ENSG00000102048_ASB9	0	1	−1.87
ENSG00000249307_LINC01088	0	1	−1.87
ENSG00000197956_S100A6	0	1	−1.86
ENSG00000072274_TFRC	0	1	−1.86
ENSG00000120669_SOHLH2	0	1	−1.85
ENSG00000179431_FJX1	0	1	−1.85
ENSG00000138336_TET1	0	1	−1.85
ENSG00000103202_NME4	0	1	−1.85
ENSG00000136928_GABBR2	0	1	−1.85
ENSG00000119138_KLF9	0	1	−1.85
ENSG00000111875_ASF1A	0	1	−1.84

ENSG00000272716_RP11-563N4.1	0	1	−1.83
ENSG00000169884_WNT10B	0	1	−1.83
ENSG00000147168_IL2RG	0	1	−1.83
ENSG00000146416_AIG1	0	1	−1.83
ENSG00000187772_LIN28B	0	1	−1.82
ENSG00000164199_GPR98	0	1	−1.82
ENSG00000113356_POLR3G	0	1	−1.81
ENSG00000254319_RP11-134O21.1	0	1	−1.81
ENSG00000134802_SLC43A3	0	1	−1.80
ENSG00000183010_PYCR1	0	1	−1.79
ENSG00000143321_HDGF	0	1	−1.79
ENSG00000183032_SLC25A21	0	1	−1.79
ENSG00000090861_AARS	0	1	−1.79
ENSG00000162039_MEIOB	0	1	−1.79
ENSG00000168497_SDPR	0	1	−1.79
ENSG00000027075_PRKCH	0	1	−1.78
ENSG00000165238_WNK2	0	1	−1.77
ENSG00000026508_CD44	0	1	−1.77
ENSG00000136040_PLXNC1	0	1	−1.77
ENSG00000189046_ALKBH2	0	1	−1.76
ENSG00000106034_CPED1	0	1	−1.76
ENSG00000260266_CTD-2311M21.2	0	1	−1.75
ENSG00000187997_C17orf99	0	1	−1.75
ENSG00000120913_PDLIM2	0	1	−1.75
ENSG00000244274_DBNDD2	0	1	−1.75
ENSG00000266709_RP11-214O1.2	0	1	−1.74
ENSG00000181472_ZBTB2	0	1	−1.74
ENSG00000260708_CTA-29F11.1	0	1	−1.74
ENSG00000187134_AKR1C1	0	1	−1.74
ENSG00000185885_IFITM1	0	1	−1.73
ENSG00000157657_ZNF618	0	1	−1.73
ENSG00000124019_FAM124B	0	1	−1.73
ENSG00000240280_TCAM1P	0	1	−1.73
ENSG00000239415_AP001469.9	0	1	−1.72
ENSG00000168754_FAM178B	0	1	−1.72
ENSG00000226950_DANCR	0	1	−1.72
ENSG00000148803_FUOM	0	1	−1.71
ENSG00000185760_KCNQ5	0	1	−1.71
ENSG00000181444_ZNF467	0	1	−1.71
ENSG00000225373_WASH5P	0	1	−1.71
ENSG00000137309_HMGA1	0	1	−1.70

ENSG00000165060_FXN	0	1	−1.70
ENSG00000169946_ZFPM2	0	1	−1.70
ENSG00000184900_SUMO3	0	1	−1.70
ENSG00000167601_AXL	0	1	−1.69
ENSG00000232445_RP11-132A1.4	0	1	−1.69
ENSG00000175538_KCNE3	0	1	−1.68
ENSG00000156206_C15orf26	0	1	−1.68
ENSG00000004478_FKBP4	0	1	−1.68
ENSG00000060982_BCAT1	0	1	−1.68
ENSG00000181274_FRAT2	0	1	−1.67
ENSG00000166902_MRPL16	0	1	−1.67
ENSG00000214049_UCA1	0	1	−1.67
ENSG00000132749_MTL5	0	1	−1.67
ENSG00000168209_DDIT4	0	1	−1.67
ENSG00000144130_NT5DC4	0	1	−1.67
ENSG00000260065_CTA-445C9.15	0	1	−1.67
ENSG00000166825_ANPEP	0	1	−1.66
ENSG00000149485_FADS1	0	1	−1.66
ENSG00000248275_TRIM52-AS1	0	1	−1.65
ENSG00000118513_MYB	0	1	−1.65
ENSG00000160200_CBS	0	1	−1.65
ENSG00000169679_BUB1	0	1	−1.65
ENSG00000169220_RGS14	0	1	−1.65
ENSG00000205639_MFSD2B	0	1	−1.65
ENSG00000087077_TRIP6	0	1	−1.65
ENSG00000184058_TBX1	0	1	−1.65
ENSG00000129991_TNNI3	0	1	−1.65
ENSG00000115183_TANC1	0	1	−1.65
ENSG00000145220_LYAR	0	1	−1.64
ENSG00000264198_RP11-94L15.2	0	1	−1.64
ENSG00000213178_RP11-641D5.1	0	1	−1.64
ENSG00000134762_DSC3	0	1	−1.63
ENSG00000144668_ITGA9	0	1	−1.63
ENSG00000249992_TMEM158	0	1	−1.62
ENSG00000169750_RAC3	0	1	−1.62
ENSG00000165905_GYLTL1B	0	1	−1.62
ENSG00000225485_ARHGAP23	0	1	−1.61
ENSG00000120254_MTHFD1L	0	1	−1.60
ENSG00000169710_FASN	0	1	−1.60
ENSG00000170515_PA2G4	0	1	−1.60
ENSG00000099958_DERL3	0	1	−1.60

ENSG00000113369_ARRDC3	0	1	−1.60
ENSG00000185811_IKZF1	0	1	−1.60
ENSG00000172752_COL6A5	0	1	−1.60
ENSG00000265933_LINC00668	0	1	−1.60
ENSG00000163072_NOSTRIN	0	1	−1.59
ENSG00000163584_RPL22L1	0	1	−1.59
ENSG00000160867_FGFR4	0	1	−1.59
ENSG00000142178_SIK1	0	1	−1.59
ENSG00000185559_DLK1	0	1	−1.59
ENSG00000272452_RP11-391M1.4	0	1	−1.58
ENSG00000185269_NOTUM	0	1	−1.58
ENSG00000155307_SAMSN1	0	1	−1.58
ENSG00000166197_NOLC1	0	1	−1.58
ENSG00000184675_AMER1	0	1	−1.58
ENSG00000141384_TAF4B	0	1	−1.57
ENSG00000239672_NME1	0	1	−1.57
ENSG00000235335_AC016723.4	0	1	−1.57
ENSG00000247095_MIR210HG	0	1	−1.57
ENSG00000140905_GCSH	0	1	−1.57
ENSG00000116990_MYCL	0	1	−1.57
ENSG00000114631_PODXL2	0	1	−1.57
ENSG00000227518_MIR1302-9	0	1	−1.57
ENSG00000120129_DUSP1	0	1	−1.56
ENSG00000020577_SAMD4A	0	1	−1.56
ENSG00000150764_DIXDC1	0	1	−1.56
ENSG00000132541_HRSP12	0	1	−1.56
ENSG00000138449_SLC40A1	0	1	−1.55
ENSG00000238164_RP3-395M20.8	0	1	−1.55
ENSG00000147454_SLC25A37	0	1	−1.55
ENSG00000125731_SH2D3A	0	1	−1.55
ENSG00000115107_STEAP3	0	1	−1.54
ENSG00000101187_SLCO4A1	0	1	−1.54
ENSG00000153443_UBALD1	0	1	−1.54
ENSG00000160124_CCDC58	0	1	−1.54
ENSG00000128050_PAICS	0	1	−1.54
ENSG00000143630_HCN3	0	1	−1.54
ENSG00000111907_TPD52L1	0	1	−1.54
ENSG00000140350_ANP32A	0	1	−1.54
ENSG00000139146_FAM60A	0	1	−1.54
ENSG00000124942_AHNAK	0	1	−1.53
ENSG00000164687_FABP5	0	1	−1.53

ENSG00000138442_WDR12	0	1	−1.53
ENSG00000180537_RNF182	0	1	−1.53
ENSG00000136261_BZW2	0	1	−1.52
ENSG00000173334_TRIB1	0	1	−1.52
ENSG00000137876_RSL24D1	0	1	−1.52
ENSG00000141068_KSR1	0	1	−1.52
ENSG00000146530_VWDE	0	1	−1.52
ENSG00000242125_SNHG3	0	1	−1.52
ENSG00000008394_MGST1	0	1	−1.51
ENSG00000083828_ZNF586	0	1	−1.51
ENSG00000007038_PRSS21	0	1	−1.51
ENSG00000172059_KLF11	0	1	−1.51
ENSG00000270696_RP11-342K6.1	0	1	−1.51
ENSG00000149781_FERMT3	0	1	−1.51
ENSG00000214960_ISPD	0	1	−1.51
ENSG00000110172_CHORDC1	0	1	−1.50
ENSG00000127507_EMR2	0	1	−1.50
ENSG00000170689_HOXB9	0	1	−1.50
ENSG00000080824_HSP90AA1	0	1	−1.50
ENSG00000136938_ANP32B	0	1	−1.50
ENSG00000164877_MICALL2	0	1	−1.50
ENSG00000112208_BAG2	0	1	−1.50
ENSG00000162496_DHRS3	0	1	−1.50
ENSG00000205978_NYNRIN	0	1	−1.49
ENSG00000100479_POLE2	0	1	−1.49
ENSG00000174004_NRROS	0	1	−1.49
ENSG00000215146_RP11-313J2.1	0	1	−1.49
ENSG00000079308_TNS1	0	1	−1.49
ENSG00000147251_DOCK11	0	1	−1.49
ENSG00000179388_EGR3	0	1	−1.49
ENSG00000135069_PSAT1	0	1	−1.49
ENSG00000165732_DDX21	0	1	−1.48
ENSG00000203989_RHOXF2B	0	1	−1.48
ENSG00000181163_NPM1	0	1	−1.48
ENSG00000143595_AQP10	0	1	−1.48
ENSG00000115935_WIPF1	0	1	−1.48
ENSG00000171316_CHD7	0	1	−1.48
ENSG00000187514_PTMA	0	1	−1.48
ENSG00000167994_RAB3IL1	0	1	−1.48
ENSG00000111906_HDDC2	0	1	−1.48
ENSG00000104691_UBXN8	0	1	−1.48

ENSG00000113739_STC2	0	1	−1.48
ENSG00000126561_STAT5A	0	1	−1.48
ENSG00000170004_CHD3	0	1	−1.48
ENSG00000203875_SNHG5	0	1	−1.47
ENSG00000176597_B3GNT5	0	1	−1.47
ENSG00000105821_DNAJC2	0	1	−1.47
ENSG00000119950_MXI1	0	1	−1.47
ENSG00000184916_JAG2	0	1	−1.47
ENSG00000112159_MDN1	0	1	−1.47
ENSG00000126368_NR1D1	0	1	−1.47
ENSG00000173726_TOMM20	0	1	−1.47
ENSG00000203668_CHML	0	1	−1.47
ENSG00000070814_TCOF1	0	1	−1.47
ENSG00000136830_FAM129B	0	1	−1.46
ENSG00000248837_RP11-412P11.1	0	1	−1.46
ENSG00000224652_LINC00885	0	1	−1.46
ENSG00000138650_PCDH10	0	1	−1.46
ENSG00000167641_PPP1R14A	0	1	−1.46
ENSG00000240972_MIF	0	1	−1.46
ENSG00000134333_LDHA	0	1	−1.46
ENSG00000108559_NUP88	0	1	−1.46
ENSG00000144381_HSPD1	0	1	−1.46
ENSG00000108561_C1QBP	0	1	−1.45
ENSG00000138050_THUMPD2	0	1	−1.45
ENSG00000258232_RP11-161H23.5	0	1	−1.45
ENSG00000153574_RPIA	0	1	−1.45
ENSG00000196950_SLC39A10	0	1	−1.45
ENSG00000181218_HIST3H2A	0	1	−1.45
ENSG00000177910_SPATA31C2	0	1	−1.45
ENSG00000181773_GPR3	0	1	−1.45
ENSG00000166394_CYB5R2	0	1	−1.45
ENSG00000159399_HK2	0	1	−1.45
ENSG00000187699_C2orf88	0	1	−1.45
ENSG00000128714_HOXD13	0	1	−1.45
ENSG00000140993_TIGD7	0	1	−1.44
ENSG00000198807_PAX9	0	1	−1.44
ENSG00000262251_RP11-199F11.2	0	1	−1.44
ENSG00000266872_RP11-19P22.8	0	1	−1.44
ENSG00000205212_CCDC144NL	0	1	−1.44
ENSG00000233461_RP11-295G20.2	0	1	−1.44
ENSG00000120162_MOB3B	0	1	−1.44

ENSG00000168461_RAB31	0	1	−1.44
ENSG00000267321_RP11-1094M14.11	0	1	−1.44
ENSG00000109519_GRPEL1	0	1	−1.43
ENSG00000269600_AC016629.3	0	1	−1.43
ENSG00000164620_RELL2	0	1	−1.43
ENSG00000179241_LDLRAD3	0	1	−1.43
ENSG00000182118_FAM89A	0	1	−1.42
ENSG00000126457_PRMT1	0	1	−1.42
ENSG00000144840_RABL3	0	1	−1.42
ENSG00000267059_UQCR11	0	1	−1.42
ENSG00000162377_COA7	0	1	−1.42
ENSG00000144895_EIF2A	0	1	−1.42
ENSG00000221944_TIGD1	0	1	−1.42
ENSG00000198015_MRPL42	0	1	−1.42
ENSG00000254726_MEX3A	0	1	−1.42
ENSG00000221823_PPP3R1	0	1	−1.42
ENSG00000156970_BUB1B	0	1	−1.42
ENSG00000171421_MRPL36	0	1	−1.42
ENSG00000100889_PCK2	0	1	−1.42
ENSG00000154153_FAM134B	0	1	−1.42
ENSG00000165716_FAM69B	0	1	−1.42
ENSG00000160193_WDR4	0	1	−1.41
ENSG00000140455_USP3	0	1	−1.41
ENSG00000151779_NBAS	0	1	−1.41
ENSG00000114850_SSR3	0	1	−1.41
ENSG00000197498_RPF2	0	1	−1.41
ENSG00000100321_SYNGR1	0	1	−1.41
ENSG00000270170_NCBP2-AS2	0	1	−1.41
ENSG00000141101_NOB1	0	1	−1.41
ENSG00000132423_COQ3	0	1	−1.41
ENSG00000111863_ADTRP	0	1	−1.41
ENSG00000254682_RP11-660L16.2	0	1	−1.41
ENSG00000152620_NADK2	0	1	−1.40
ENSG00000099256_PRTFDC1	0	1	−1.40
ENSG00000083807_SLC27A5	0	1	−1.40
ENSG00000114942_EEF1B2	0	1	−1.40
ENSG00000171282_RP11-1055B8.7	0	1	−1.40
ENSG00000107949_BCCIP	0	1	−1.40
ENSG00000196305_IARS	0	1	−1.40
ENSG00000215784_FAM72D	0	1	−1.40
ENSG00000116161_CACYBP	0	1	−1.40

ENSG00000003249_DBNDD1	0	1	−1.39
ENSG00000196365_LONP1	0	1	−1.39
ENSG00000106852_LHX6	0	1	−1.39
ENSG00000157404_KIT	0	1	−1.39
ENSG00000124802_EEF1E1	0	1	−1.39
ENSG00000168404_MLKL	0	1	−1.39
ENSG00000272841_RP3-428L16.2	0	1	−1.39
ENSG00000222041_LINC00152	0	1	−1.39
ENSG00000147804_SLC39A4	0	1	−1.39
ENSG00000198157_HMGN5	0	1	−1.39
ENSG00000175110_MRPS22	0	1	−1.38
ENSG00000236279_CLEC2L	0	1	−1.38
ENSG00000073792_IGF2BP2	0	1	−1.38
ENSG00000162543_UBXN10	0	1	−1.38
ENSG00000104408_EIF3E	0	1	−1.38
ENSG00000141569_TRIM65	0	1	−1.38
ENSG00000138035_PNPT1	0	1	−1.38
ENSG00000114126_TFDP2	0	1	−1.38
ENSG00000112077_RHAG	0	1	−1.38
ENSG00000218226_TATDN2P2	2.42E−11	1	−1.38
ENSG00000072571_HMMR	0	1	−1.38
ENSG00000135549_PKIB	0	1	−1.37
ENSG00000179598_PLD6	0	1	−1.37
ENSG00000100351_GRAP2	0	1	−1.37
ENSG00000171490_RSL1D1	0	1	−1.37
ENSG00000196526_AFAP1	0	1	−1.37
ENSG00000225339_RP11-513I15.6	0	1	−1.37
ENSG00000102287_GABRE	0	1	−1.37
ENSG00000112996_MRPS30	0	1	−1.37
ENSG00000115541_HSPE1	0	1	−1.37
ENSG00000113649_TCERG1	0	1	−1.37
ENSG00000262814_MRPL12	0	1	−1.37
ENSG00000162599_NFIA	0	1	−1.36
ENSG00000177410_ZFAS1	0	1	−1.36
ENSG00000242284_CT45A5	0	1	−1.36
ENSG00000163818_LZTFL1	0	1	−1.36
ENSG00000133773_CCDC59	0	1	−1.36
ENSG00000215695_RSC1A1	0	1	−1.36
ENSG00000198728_LDB1	0	1	−1.36
ENSG00000166788_SAAL1	0	1	−1.36
ENSG00000205777_GAGE1	0	1	−1.36

ENSG00000072071_LPHN1	0	1	−1.36
ENSG00000249196_RP11-669N7.2	0	1	−1.36
ENSG00000197465_GYPE	0	1	−1.35
ENSG00000173638_SLC19A1	0	1	−1.35
ENSG00000079335_CDC14A	0	1	−1.35
ENSG00000106211_HSPB1	0	1	−1.35
ENSG00000100219_XBP1	0	1	−1.35
ENSG00000090447_TFAP4	0	1	−1.35
ENSG00000140459_CYP11A1	0	1	−1.35
ENSG00000159131_GART	0	1	−1.35
ENSG00000112972_HMGCS1	0	1	−1.35
ENSG00000234769_WASH4P	0	1	−1.35
ENSG00000162738_VANGL2	0	1	−1.35
ENSG00000215861_WI2-1896O14.1	1.50E−12	1	−1.35
ENSG00000185101_ANO9	0	1	−1.35
ENSG00000103356_EARS2	0	1	−1.35
ENSG00000153885_KCTD15	0	1	−1.35
ENSG00000065057_NTHL1	0	1	−1.34
ENSG00000162433_AK4	0	1	−1.34
ENSG00000138363_ATIC	0	1	−1.34
ENSG00000126602_TRAP1	0	1	−1.34
ENSG00000198805_PNP	0	1	−1.34
ENSG00000250479_CHCHD10	0	1	−1.34
ENSG00000115364_MRPL19	0	1	−1.34
ENSG00000151012_SLC7A11	0	1	−1.34
ENSG00000160284_SPATC1L	0	1	−1.34
ENSG00000196550_FAM72A	0	1	−1.34
ENSG00000165449_SLC16A9	0	1	−1.34
ENSG00000071575_TRIB2	0	1	−1.34
ENSG00000005187_ACSM3	0	1	−1.34
ENSG00000203817_FAM72C	0	1	−1.34
ENSG00000109971_HSPA8	0	1	−1.34
ENSG00000163811_WDR43	0	1	−1.34
ENSG00000162402_USP24	0	1	−1.34
ENSG00000171813_PWWP2B	0	1	−1.34
ENSG00000105610_KLF1	0	1	−1.34
ENSG00000183077_AFMID	0	1	−1.33
ENSG00000101911_PRPS2	0	1	−1.33
ENSG00000230844_ZNF674-AS1	0	1	−1.33
ENSG00000196155_PLEKHG4	0	1	−1.33
ENSG00000166582_CENPV	0	1	−1.33

ENSG00000147955_SIGMAR1	0	1	−1.33
ENSG00000113460_BRIX1	0	1	−1.33
ENSG00000065427_KARS	0	1	−1.33
ENSG00000241732_RP11-38P22.2	0	1	−1.33
ENSG00000147905_ZCCHC7	0	1	−1.33
ENSG00000106105_GARS	0	1	−1.33
ENSG00000105676_ARMC6	0	1	−1.33
ENSG00000165644_COMTD1	0	1	−1.33
ENSG00000067533_RRP15	0	1	−1.32
ENSG00000179715_PCED1B	0	1	−1.32
ENSG00000228463_AP006222.2	0	1	−1.32
ENSG00000146410_MTFR2	0	1	−1.32
ENSG00000069998_CECR5	0	1	−1.32
ENSG00000135521_LTV1	0	1	−1.32
ENSG00000237506_RPSAP15	0	1	−1.32
ENSG00000035141_FAM136A	0	1	−1.32
ENSG00000101935_AMMECR1	0	1	−1.32
ENSG00000214110_LDHAP4	0	1	−1.32
ENSG00000134480_CCNH	0	1	−1.32
ENSG00000056736_IL17RB	0	1	−1.32
ENSG00000224287_MSL3P1	0	1	−1.32
ENSG00000180817_PPA1	0	1	−1.32
ENSG00000136897_MRPL50	0	1	−1.32
ENSG00000164818_HEATR2	0	1	−1.32
ENSG00000079462_PAFAH1B3	0	1	−1.31
ENSG00000131721_RHOXF2	0	1	−1.31
ENSG00000116750_UCHL5	0	1	−1.31
ENSG00000188610_FAM72B	0	1	−1.31
ENSG00000167721_TSR1	0	1	−1.31
ENSG00000109255_NMU	0	1	−1.31
ENSG00000120963_ZNF706	0	1	−1.31
ENSG00000067334_DNTTIP2	0	1	−1.30
ENSG00000106591_MRPL32	0	1	−1.30
ENSG00000137720_C11orf1	0	1	−1.30
ENSG00000122140_MRPS2	0	1	−1.30
ENSG00000149591_TAGLN	0	1	1.30
ENSG00000127328_RAB3IP	0	1	1.30
ENSG00000152926_ZNF117	0	1	1.30
ENSG00000062524_LTK	0	1	1.31
ENSG00000102879_CORO1A	0	1	1.32
ENSG00000198736_MSRB1	0	1	1.33

ENSG00000160223_ICOSLG	0	1	1.33
ENSG00000128284_APOL3	0	1	1.33
ENSG00000221963_APOL6	0	1	1.34
ENSG00000183628_DGCR6	0	1	1.35
ENSG00000084731_KIF3C	0	1	1.36
ENSG00000136717_BIN1	0	1	1.36
ENSG00000165475_CRYL1	0	1	1.36
ENSG00000272068_RP11-284F21.9	0	1	1.36
ENSG00000100379_KCTD17	0	1	1.36
ENSG00000106479_ZNF862	0	1	1.38
ENSG00000136943_CTSV	0	1	1.39
ENSG00000101986_ABCD1	0	1	1.39
ENSG00000149212_SESN3	0	1	1.40
ENSG00000124104_SNX21	0	1	1.40
ENSG00000158856_DMTN	0	1	1.41
ENSG00000183087_GAS6	0	1	1.41
ENSG00000189120_SP6	0	1	1.42
ENSG00000144648_ACKR2	0	1	1.43
ENSG00000049239_H6PD	0	1	1.43
ENSG00000089692_LAG3	0	1	1.43
ENSG00000125148_MT2A	0	1	1.45
ENSG00000104154_SLC30A4	0	1	1.48
ENSG00000075399_VPS9D1	0	1	1.48
ENSG00000254837_AP001372.2	0	1	1.49
ENSG00000090238_YPEL3	0	1	1.49
ENSG00000261342_AC006538.1	0	1	1.50
ENSG00000225138_CTD-2228K2.7	0	1	1.50
ENSG00000121064_SCPEP1	0	1	1.51
ENSG00000160325_CACFD1	0	1	1.51
ENSG00000135862_LAMC1	0	1	1.51
ENSG00000196622_RIMBP3	0	1	1.51
ENSG00000185015_CA13	0	1	1.51
ENSG00000165272_AQP3	0	1	1.52
ENSG00000172380_GNG12	0	1	1.52
ENSG00000135482_ZC3H10	0	1	1.52
ENSG00000009950_MLXIPL	0	1	1.53
ENSG00000159314_ARHGAP27	0	1	1.53
ENSG00000137460_FHDC1	0	1	1.54
ENSG00000175482_POLD4	0	1	1.55
ENSG00000222009_BTBD19	0	1	1.56
ENSG00000114626_ABTB1	0	1	1.56

ENSG00000235750_KIAA0040	0	1	1.57
ENSG00000178809_TRIM73	0	1	1.58
ENSG00000118762_PKD2	0	1	1.58
ENSG00000020181_GPR124	0	1	1.58
ENSG00000176834_VSIG10	0	1	1.59
ENSG00000114737_CISH	0	1	1.59
ENSG00000253882_RP11-61L23.2	0	1	1.63
ENSG00000267013_CTD-2171N6.1	0	1	1.65
ENSG00000143153_ATP1B1	0	1	1.65
ENSG00000177548_RABEP2	0	1	1.65
ENSG00000178718_RPP25	0	1	1.66
ENSG00000188818_ZDHHC11	0	1	1.68
ENSG00000092964_DPYSL2	0	1	1.69
ENSG00000232434_C9orf172	0	1	1.70
ENSG00000082014_SMARCD3	0	1	1.71
ENSG00000170379_FAM115C	0	1	1.72
ENSG00000197381_ADARB1	0	1	1.72
ENSG00000197093_GAL3ST4	0	1	1.75
ENSG00000099994_SUSD2	0	1	1.76
ENSG00000167552_TUBA1A	0	1	1.76
ENSG00000182179_UBA7	0	1	1.76
ENSG00000146021_KLHL3	0	1	1.77
ENSG00000073712_FERMT2	0	1	1.78
ENSG00000104081_BMF	0	1	1.78
ENSG00000237037_NDUFA6-AS1	0	1	1.79
ENSG00000135622_SEMA4F	0	1	1.79
ENSG00000119242_CCDC92	0	1	1.79
ENSG00000102057_KCND1	0	1	1.79
ENSG00000121797_CCRL2	0	1	1.80
ENSG00000116525_TRIM62	0	1	1.81
ENSG00000110002_VWA5A	0	1	1.82
ENSG00000106665_CLIP2	0	1	1.83
ENSG00000109610_SOD3	0	1	1.83
ENSG00000197380_DACT3	0	1	1.84
ENSG00000071246_VASH1	0	1	1.84
ENSG00000005379_BZRAP1	0	1	1.84
ENSG00000142459_EVI5L	0	1	1.85
ENSG00000151726_ACSL1	0	1	1.87
ENSG00000112297_AIM1	0	1	1.87
ENSG00000160781_PAQR6	0	1	1.89
ENSG00000063438_AHRR	0	1	1.89

ENSG00000117318_ID3	0	1	1.89
ENSG00000164938_TP53INP1	0	1	1.89
ENSG00000230487_PSMG3-AS1	0	1	1.89
ENSG00000166311_SMPD1	0	1	1.90
ENSG00000228314_CYP4F29P	0	1	1.91
ENSG00000239521_GATS	0	1	1.92
ENSG00000104419_NDRG1	0	1	1.93
ENSG00000140678_ITGAX	0	1	1.94
ENSG00000124357_NAGK	0	1	1.96
ENSG00000141504_SAT2	0	1	1.98
ENSG00000129646_QRICH2	0	1	1.98
ENSG00000161149_TUBA3FP	0	1	2.00
ENSG00000168140_VASN	0	1	2.01
ENSG00000007516_BAIAP3	0	1	2.04
ENSG00000159640_ACE	0	1	2.04
ENSG00000113916_BCL6	0	1	2.06
ENSG00000125968_ID1	0	1	2.07
ENSG00000168938_PPIC	0	1	2.08
ENSG00000135924_DNAJB2	0	1	2.08
ENSG00000099957_P2RX6	0	1	2.12
ENSG00000114270_COL7A1	0	1	2.14
ENSG00000148180_GSN	0	1	2.15
ENSG00000181625_SLX1B	0	1	2.16
ENSG00000258947_TUBB3	0	1	2.31
ENSG00000187634_SAMD11	0	1	2.31
ENSG00000001617_SEMA3F	0	1	2.36
ENSG00000124762_CDKN1A	0	1	2.38
ENSG00000010404_IDS	0	1	2.42
ENSG00000197694_SPTAN1	0	1	2.42
ENSG00000230606_AC159540.1	0	1	2.44
ENSG00000133216_EPHB2	0	1	2.45
ENSG00000213977_TAX1BP3	0	1	2.45
ENSG00000105290_APLP1	0	1	2.55
ENSG00000258839_MC1R	0	1	2.80
ENSG00000176485_PLA2G16	0	1	3.07
ENSG00000185338_SOCS1	0	1	3.19
ENSG00000184557_SOCS3	0	1	3.25

PPDE, posterior probability of differential expression; PPEE, posterior probability of equal expression; FC, fold change.

The table presents a list of genes with PPDE = 1 and $|\log_2 FC| > 1.3$.

Table S3. Pathway enrichment analysis of differentially expressed genes between the control and rosuvastatin-treated groups.

Pathway name	Set size	Candidates contained	p-value	q-value	Pathway source
Validated targets of C-MYC transcriptional activation	89	12 (13.5%)	1.31e−05	0.0134	PID
Cytosolic tRNA aminoacylation	24	6 (25.0%)	5.95e−05	0.0152	Reactome
Hematopoietic stem cell differentiation	48	8 (16.7%)	8.01e−05	0.0152	Wikipathways
Erythrocytes take up carbon dioxide and release oxygen	9	4 (44.4%)	8.91e−05	0.0152	Reactome
Erythrocytes take up oxygen and release carbon dioxide	9	4 (44.4%)	8.91e−05	0.0152	Reactome
O_2/CO_2 exchange in erythrocytes	9	4 (44.4%)	8.91e−05	0.0152	Reactome
tRNA aminoacylation	42	7 (16.7%)	2.25e−04	0.0329	Reactome
Folate metabolism	31	3 (19.4%)	2.73e−04	0.0349	INOH

PID, Pathway Interaction Database; INOH, Integrating Network Objects with Hierarchies.

Of the 607 genes in the input list, 336 (62.1%) were present in at least one pathway. False discovery rate < 0.05.

Table S4. List of candidate genes that overlap with those determined in the pathway enrichment analysis using DAVID.

C-Myc pathway									
**Gene symbol**	**Imatinib**			**Rosuvastatin**			**Imatinib/Rosuvastatin**		
	**Log2 fold change**	**Control mean**	**Treated mean**	**Log2 fold change**	**Control mean**	**Treated mean**	**Log2 fold change**	**Control mean**	**Treated mean**
FOSL1	−3.74511	590.6308	44.04782	−1.94846	835.8824	216.5711	−5.43579	795.8823	18.38714
NPM1	−1.5447	50050.84	17155.78	−1.17222	70833.78	31431.56	−2.13594	67444.12	15344.85
LDHA	−2.04913	28101.32	6790.1	−1.24005	39770.02	16836.98	−2.41647	37866.88	7092.969
TFRC	−1.26245	107091.6	44639.71	−2.09212	151560	35546.2	−3.69737	144307.3	11124.22
TAF4B	−1.82832	1029.79	289.9815	−0.96551	1457.396	746.3275	−2.61332	1387.655	226.7747
NME1	−2.43546	5484.41	1013.871	−1.745	7761.738	2315.593	−4.05514	7390.31	444.5738
HSPD1	−1.64236	25645.61	8215.093	−0.98942	36294.61	18280.87	−2.50293	34557.78	6096.597
PTMA	−1.36171	26854.08	10449.45	−1.39235	38004.87	14477.72	−2.71104	36186.2	5526.357
LIN28B	−2.14453	3006.551	679.9882	−1.36763	4254.98	1648.923	−2.74892	4051.364	602.6896
BCAT1	−1.63939	7144.235	2293.24	−1.15919	10110.78	4527.245	−2.12624	9626.945	2205.095
HMGA1	−1.42446	21648.47	8065.34	−1.81046	30637.71	8734.82	−2.51598	29171.58	5100.048
HSP90AA1	−1.38254	81158.19	31127.65	−0.83725	114858	64287.21	−2.38162	109361.7	20985.84
*TERT**	−2.84713	554.6699	77.08369	−2.58332	784.9892	130.9802	−5.93012	747.4245	12.25809
MYC	−2.16708	12780.29	2845.673	−0.59651	18087.14	11961.99	−2.34658	17221.6	3385.958

Hematopoietic stem cell differentiation pathway									
**Gene symbol**	**Imatinib**			**Rosuvastatin**			**Imatinib/Rosuvastatin**		
	**Log2 fold change**	**Control mean**	**Treated mean**	**Log2 fold change**	**Control mean**	**Treated mean**	**Log2 fold change**	**Control mean**	**Treated mean**
IKZF1	−0.49211	2120.604	1507.72	−1.25745	3001.157	1255.335	−1.59915	2857.54	943.1922
GYPA	−0.44834	8775.542	6431.45	−2.79274	12419.47	1792.269	−1.55808	11825.15	4015.867
KCNH2	−1.64838	27771.63	8859.118	−2.31195	39303.44	7915.222	−3.16163	37422.62	4182.053
LMO2	−0.12414	4500.563	4129.483	−2.34159	6369.362	1256.631	−2.08222	6064.564	1432.154

KLF1	0.029554	5287.344	5396.776	−1.55884	7482.844	2539.848	−1.33052	7124.762	2832.982
MYB	−2.16855	7706.533	1714.194	−1.12634	10906.57	4996.05	−2.75816	10384.65	1534.986
MXI1	−0.45956	654.9246	476.2671	−0.9616	926.8732	475.9378	−0.73983	882.5189	528.4601
STAT5A	−1.54426	10604.6	3636.019	−1.43154	15008.02	5564.005	−2.15185	14289.83	3215.564

* *TERT* was detected when median normalization was used (≥100).
